# Phenotypical screening on metastatic PRCC-TFE3 fusion translocation renal cell carcinoma organoids reveals potential therapeutic agents

**DOI:** 10.1007/s12094-021-02774-8

**Published:** 2022-02-03

**Authors:** Chuanzhen Cao, Xiaomei Lan, Bingqing Shang, Weixing Jiang, Lei Guo, Shan Zheng, Xingang Bi, Aiping Zhou, Zhijian Sun, Jianzhong Shou

**Affiliations:** 1 Department of Urology, National Cancer Center/National Clinical Research Center for Cancer/Cancer Hospital, Chinese Academy of Medical Sciences and Peking Union Medical College, Panjiayuan Nanli 17#, Chaoyang District, Beijing, 100021 People’s Republic of China; 2 K2 Oncology Co. Ltd., Beijing, 100176 People’s Republic of China; 3 Department of Pathology, National Cancer Center/National Clinical Research Center for Cancer/Cancer Hospital, Chinese Academy of Medical Sciences and Peking Union Medical College, Beijing, 100021 People’s Republic of China; 4 Department of Medical Oncology, National Cancer Center/National Clinical Research Center for Cancer/Cancer Hospital, Chinese Academy of Medical Sciences and Peking Union Medical College, Panjiayuan Nanli 17#, Beijing, 100021 People’s Republic of China

**Keywords:** Translocation renal cell carcinoma, Metastatic, Organoid, High-throughput screening

## Abstract

**Purpose:**

Translocation renal cell carcinoma (tRCC) is a subtype that occurs predominantly in children and young individuals. Metastatic tRCC occurring in young patients is more aggressive than that occurring in older patients, and there are still no effective therapies. Organoids can mimic original tissues and be assessed by high-throughput screening (HTS). We aimed to utilize patient-derived organoids and HTS to screen drugs that can be repurposed for metastatic tRCC with PRCC-TFE3 fusion.

**Methods:**

Tumor tissues were obtained from treatment-naïve metastatic tRCC patients who underwent surgery. Histopathology and fluorescence in situ hybridization (FISH) confirmed the tRCC. Organoids derived from the dissected tissues were cultured and verified by FISH and RNA-seq. HTS was performed to seek promising drugs, and potential mechanisms were explored by RNA-seq and cell-based studies.

**Results:**

We successfully established a metastatic tRCC organoid with PRCC-TFE3 fusion, a common fusion subtype, and its characteristics were verified by histopathology, FISH, and RNA-seq. An HTS assay was developed, and the robustness was confirmed. A compound library of 1816 drugs was screened. Eventually, axitinib, crizotinib, and JQ-1 were selected for further validation and were found to induce cell cycle arrest and apoptosis. RNA-seq analyses of posttreatment organoids indicated that crizotinib induced significant changes in autophagy-related genes, consistent with the potential pathogenesis of tRCC.

**Conclusions:**

We established and validated organoids derived from tissues dissected from a patient with metastatic tRCC with PRCC-TFE3 fusion and achieved the HTS process for the first time. Crizotinib might be a targeted therapy worthy of exploration in the clinic for metastatic tRCC with PRCC-TFE3 fusion. Such organoid and HTS assays may represent a promising model system in translational research assisting in the development of clinical strategies.

**Supplementary Information:**

The online version contains supplementary material available at 10.1007/s12094-021-02774-8.

## Introduction

Microphthalmia transcription factor (MiT) family translocation renal cell carcinoma (tRCC) is a subtype of kidney cancer that occurs predominantly in children and adolescents but only accounts for 1.3 ~ 1.5% of adult RCCs [1, 2]. It has been recently defined as a distinct subset of RCC classified by characteristic morphology and clinical presentation. The unique characteristic of tRCC is the fusion of various partner genes with transcription factor E3 (TFE3), which is located on chromosome Xp11.2 [3]. The common fusion types in tRCC include PRCC-TFE3, ASPL-TFE3, and PFS-TFE3 [4]. The mechanisms underlying the oncogenic roles of TFE3 fusions in tRCC have not yet been elucidated. Previous studies indicated that TFE3 fusions could regulate autophagy and lysosomal biogenesis, which were involved in oncogenic signaling [5]. As the activity of the promoters of fusion-affected genes increases, the overexpression of fusion proteins potentiates the intrinsic oncogenic features of MiT-TFE transcription factors [3].

Pediatric tRCC is mostly indolent, while tRCC diagnosed in young adults is more aggressive with unfavorable outcomes [6, 7]. Adult patients with metastatic tRCC also present a poorer prognosis than those with non-tRCC disease [8]. However, there is no accepted standard therapy for metastatic tRCC. Therefore, understanding the functions of TFE3 fusion as an intrinsic pro-oncogenic driver might reveal potential drug targets.

Organoids are in vitro culture models of human tissues; they have a high success rate in terms of model generation and can be established quickly and retain the original tissue structure and biological information. Organoids hold great promise in the exploration and screening of potential treatment options. Over the past years, patient-derived organoids (PDOs) have been established and are becoming crucial models in basic research and drug discovery. For kidney cancer, however, only a few studies using patient-derived materials such as primary cell lines or tumor cell-derived organoids have been published about drug screening, and these studies did not involve tRCC [9, 10].

High-throughput screening (HTS) is an automated process that allows for the rapid testing of large chemical, genetic, or biological libraries. It utilizes microplates, liquid handling devices, sensitive detectors, and data-processing software to identify a small number of effectors of a particular biological mechanism from libraries.

Here, we describe the establishment of the first organoid for metastatic tRCC with a common fusion (PRCC-TFE3) and describe the HTS process with a goal of discovering effective drugs in vitro that could be further evaluated in the clinic.

## Materials and methods

### Human tumor tissues

All experiments with human tissue were approved by the Ethics Committee of the National Cancer Center/Cancer Hospital, Chinese Academy of Medical Sciences (NCC/CHCAMS) (ID: 20/246-2442), and written informed consent was obtained. Renal tumor tissue was obtained from treatment-naïve patients who underwent nephrectomy at the Department of Urology, NCC/CHCAMS. The sample was processed within 24 h after surgery. Moreover, pathological evaluation and TFE3 break-apart fluorescence in situ hybridization (FISH) detection confirmed the malignancy of the samples and the TFE3 fusion status.

### PDO culture

**Fig. 1:**
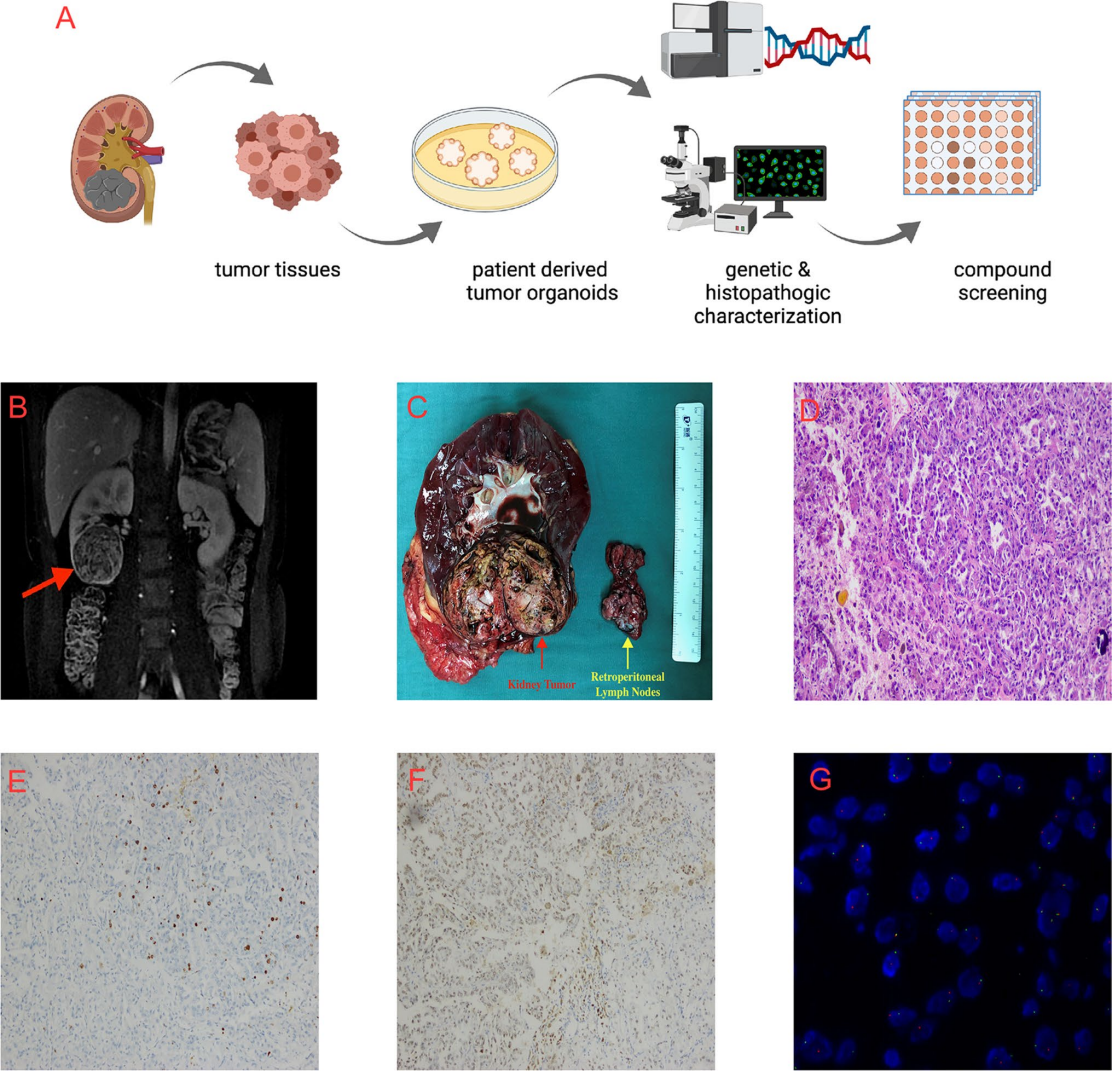
Schematic illustration and clinical feature of the metastatic PRCC-TFE3 fusion tRCC case. **A** The whole process from tissue obtain to compound screening (created by BioRender). **B** MRI indicates a solid tumor of 6.0 × 5.0 × 5.2 cm located at the lower pool of right kidney with arterial phase enhancement. **C**, The specimen picture of right kidney with a tumor located at the lower pool and the retroperitoneal lymph nodes. **D** H&E staining slide at 100 × magnification of renal cell carcinoma showed eosinophilic staining of tumor cytoplasm, papillary structure, foci of necrosis, and gravel-like calcification. **E** and **F** Immunohistochemistry of Ki-67 (5%) and TFE3 protein (positive) at 100 × magnification. **G** TFE3 break-apart FISH assay indicated the cells with split-signals accounted for 70%. Signals were considered to be split when the green and red signals were separated by a distance ≥ 2 signal diameters. Normal cells of male showed one signal of yellow while TFE3 fusion tRCC cells showed two split signals of green and red

On arrival, tumor tissue was extensively washed with cold phosphate buffered saline (PBS) and then minced into cubes smaller than 1 mm^3^ on ice. Subsequently, the minced tissue pieces were digested with collagenase (2 mg/ml, Cat. No. C9407, Sigma–Aldrich, St Louis, MO, USA) for 1 h at 37 °C and filtered through sieves with 100-μm pores. After the samples were washed twice with fresh medium (2% fetal calf serum, FCS; advanced DMEM/F 12, 2 mM HEPES, 1 × GlutaMAX-I, 200 U/ml penicillin/streptomycin) and centrifugation (300 g, 5 min), the dissociated cells were seeded into growth factor-reduced Matrigel (Corning Inc., Corning, NY, USA) in the presence of advanced DMEM/F12 at 37 °C for 30 min. Next, the surface of solidified mixture of cell suspension/Matrigel was sealed with complete human organoid medium (HOM, 1 ml), which was comprised of Advanced DMEM/F12 supplementing with series additives (1 × B27 supplement, 10 nM Lue15-Gastrin I, 1 mM N-acetylcysteine, 100 ng/ml recombinant human IGF-1, 50 ng/ml recombinant FGF-2, 20% afamin/Wnt3a conditional medium, 1 µg/ml human R-spondin, 100 ng/ml Noggin, 500 nM A-8301, 200 U/ml penicillin/streptomycin, and 10 µM Y-27632) as described by Lampis et al. [11] and Loredana et al. [12]. The medium was replaced every 3 days when the diameter of organoids ranged up to 200 ~ 300 μm, and the organoids were dissociated and passaged weekly. Matrigel was broken up by pipetting up and down several times, and organoids were collected in a tube. After centrifugation at 300 g for 5 min, organoids were dissociated using TrypLE Express (Gibco, Grand Island, NY, USA) for 10 min. Cell clusters were reseeded at a typical split ratio of 1:3. A bright-light microscope (LEICA, Wetzlar, Germany) was used for organoid bright field photographing. Early passage organoids were frozen in Recovery Cell Culture Freezing Medium (Gibco) and stored at − 80 °C before drug screening. The whole process from tissue collection to compound screening is shown in Fig. 1A. A detailed protocol will be provided by the authors upon request.

### Histology, immunohistochemistry, and TFE3 break-apart FISH assay

Tumor tissues and organoids were fixed in 4% paraformaldehyde followed by dehydration, paraffin embedding, sectioning and standard hematoxylin and eosin (H&E) staining. Immunohistochemistry (IHC) staining was performed with TFE3 (dilution-free. RMA-0663, Shandong Maixin Biotechnology Co., China), E-cadherin (dilution 1: 200. MAB-0738, Shandong Maixin Biotechnology Co., China), and Ki-67 (dilution 1: 200. GM7240, GeneTech Co., Ltd., Shanghai, China) antibodies on an Autostainer 480S (Fa Medac). Images of stained samples were taken with an Olympus DP73 microscope (Olympus, Japan).

TFE3 protein expression confirmed by IHC is routinely used for preliminary diagnosis, but clinically FISH is the gold standard for diagnosis of TFE3 fusion tRCC [13]. A dual-color, break-apart FISH assay was performed to detect TFE3 using the TFE3 (Xp11.2) break probe set (Guangzhou LBP Medical Technology Co., Ltd., China). FISH signals were assessed under an Olympus BX51TRF microscope (Olympus, Japan). Normal cells without fusion had one (for males) or two (for females) yellow signals. Signals were considered to be split when the distance between red and green signals was ≥ 2 signal diameters. For each case, a minimum of 100 tumor nuclei were evaluated. Overlapping cells indistinguishable as separate nuclei were not included in the analysis to avoid false positives due to nuclear truncation. A positive result was defined when ≥ 15% of the tumor nuclei had split signals.

### Compound library screening

HTS was performed automatically using a mechanical liquid handling equipment mechanical arm (NAYO N96-204, NAYO Biotech. Co., Ltd. Shanghai, China). A drug library containing 1816 small molecule compounds was obtained from MedChemExpress (Shanghai, China, catalog number HY-L0022M) with modification (Supplementary Data 1). The library was reformatted into 96-well source plates with a concentration of 1 mM for automated robotic screening. In parallel, the cells were also treated with an equal volume (0.2%) of dimethyl sulfoxide (DMSO) as a negative control and 2.5 μM final BEZ-235 (MCE, Shanghai, China) as a positive control. BEZ-235 is an inhibitor of phosphatidylinositol 3-kinases (PI3-K) and is commonly used as a positive control in HTS or drug exploration [14, 15]. Plate-to-plate normalization and assay quality control were performed according to them. A 3D cell viability assay was implemented to determine the number of viable cells according to ATP level using commercially available luminescence detection reagent (CellTiter-Glo #G9683, Promega, Madison, WI). Briefly, organoids were processed as described previously [16] and plated in a 96-well low binding assay plate at a density of 8,000 cells per well in 50 μl 10% growth factor reduced Matrigel. Additional 40 μl culture medium without Matrigel was added as described above. Organoids were maintained in medium described earlier [17] and treated 2 days later by adding 10 μl culture medium with 10 μM compound to obtain a final concentration of 1 μM. The assay was terminated at day 5 by adding 50 μl CellTiter-Glo. Assay quality and robustness were evaluated with the signal window (SW) and Z factor. The SW and the Z factor have been routinely used in previous HTS studies for validation [18–20]. Triplicate wells treated with BEZ-235 and vehicle solvent (DMSO) were employed as bottom wells and top wells, respectively. When SW was above 6 and the Z factor values were between 0.44 and 0.78, the assay was qualified for HTS. The equipment of the Z factor is listed below (*σ*, standard deviation. *μ*, average. *p*, positive control. *n*, negative control) [21] as follows:

$${\rm Z} = 1 - \frac{{3 \times \left( {\sigma_{p} + \sigma_{n} } \right)}}{{\left| {\mu_{p} - \mu_{n} } \right|}}$$

### Analysis of cell cycle arrest and apoptosis

Cell cycle and apoptosis were detected as previously described [22]. Organoids were cultured and treated with the indicated drugs at different concentrations for 48 h, followed by single staining with propidium iodide (Beyotime Biotechnology Co., Ltd., China) for cell cycle analyses and double staining with propidium iodide and Annexin V-FITC (Beyotime Biotechnology Co., Ltd., China) for apoptosis analyses.

### RNA-seq analysis

Organoids were incubated with DMSO or the indicated drugs for 120 h. After harvesting, total RNA was extracted using the TraZolTM UP Plus RNA Kit. RNA was sent for sequencing and analysis (BGI Co., Ltd., Shenzhen, China). Briefly, total RNA was fragmented, and mRNA was enriched using oligo (dT) magnetic beads, followed by cDNA synthesis. Double-stranded cDNA was purified and enriched by PCR amplification, after which the library products were sequenced using BGIseq-500. The volcano plot and heatmap of differentially expressed genes (log2 of fold change > 1, *q* < 0.05) were generated by BGI using the Dr. TOM approach, a customized data mining system from BGI. Next, 232 autophagy-related genes (ARGs) were obtained from the Human Autophagy database (HADb, http://www.autophagy.lu/index.html) and used for analyses.

### Statistical analysis

Data statistical analysis was performed using Prism 6.4. The half-maximal inhibitory concentration (IC_50_) values were analyzed using nonlinear regression (curve fit). The 95% confidence interval was calculated. The differences in IC_50_ values between organoids were analyzed using the nonparametric Mann–Whitney U test. Cell cycle and apoptosis data were analyzed using Excel with Student’s *t* test. *q* < 0.05 was considered to indicate statistical significance.

## Results

### Identification of metastatic tRCC patient

One 21-year-old male patient was diagnosed with right kidney cancer and retroperitoneal lymph node metastasis. Magnetic resonance imaging (MRI) indicated a solid tumor of 6.0 × 5.0 × 5.2 cm located in the lower pool of the right kidney with arterial phase enhancement (Fig. 1B). Laparoscopic nephrectomy and retroperitoneal lymph node dissection were performed in April 2019. The pathology indicated RCC and lymph node metastasis (T1bN0M1, Stage IV) (Fig. 1C). H&E staining demonstrated that the tumor featured papillary architecture combined with foci of necrosis and gravel-like calcification (Fig. 1D). IHC showed positivity for Ki-67 and TFE3 protein expression (Fig. 1E and F). tRCC was confirmed by the FISH assay (Fig. 1G).

### Characterization of PRCC-TFE3 fusion organoids

**Fig. 2:**
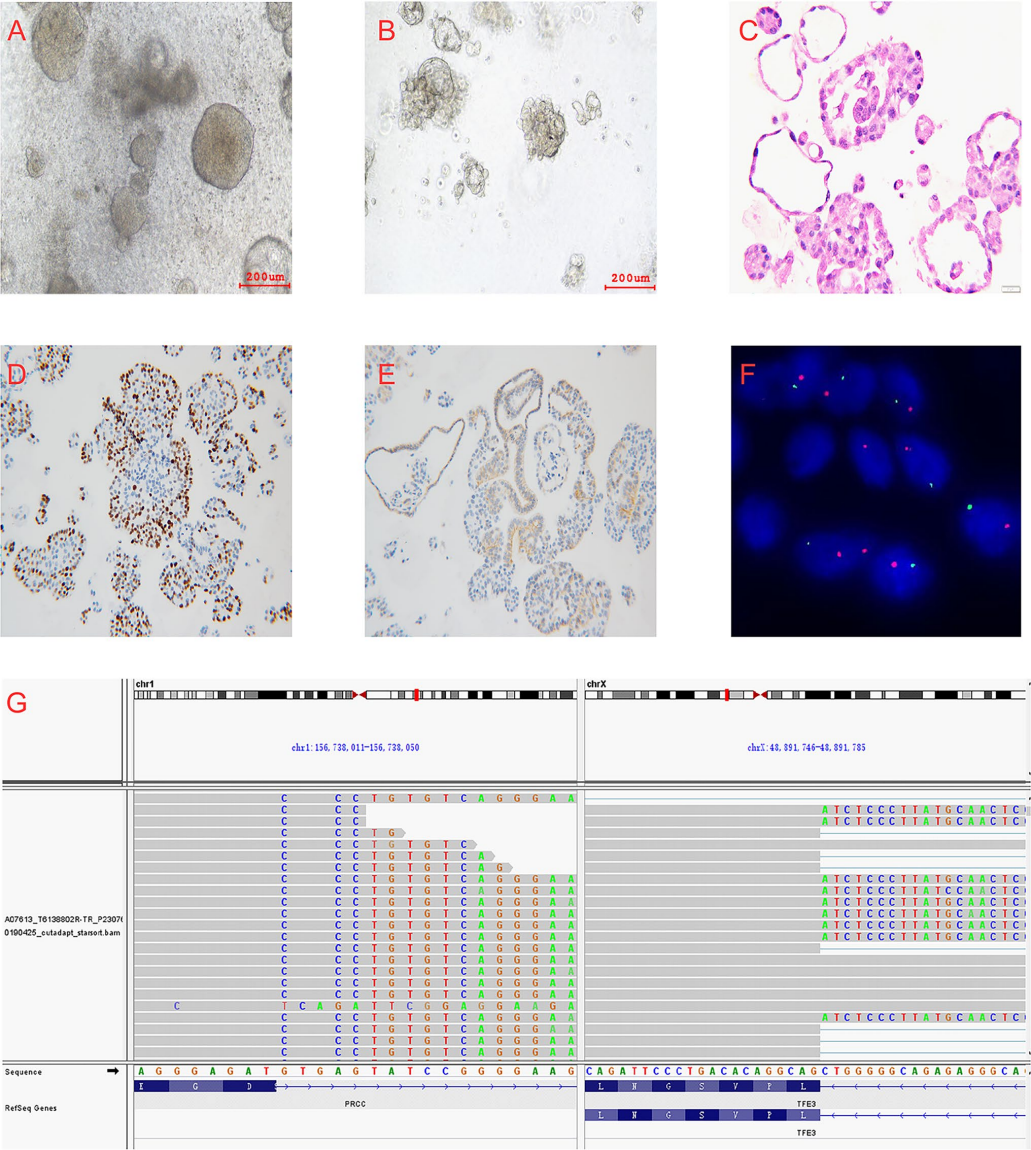
Characterization of PRCC-TFE3 fusion organoids. **A** and **B** Bright-field images of patient-derived PRCC-TFE3 fusion tRCC organoids in the early stage (passage 0) and the later stage (passage 5). **C** The H&E staining of the PRCC-TFE3 fusion tRCC organoid showed cystic phenotype with papillary architecture which has similarity with original tumor tissue at 100 × magnification. **D** Immunohistochemistry of Ki-67 (60%) at 100 × magnification of the organoid. **E** Immunohistochemistry of E-cadherin (> 50%) at 100 × magnification of the organoid. This demonstrated a clear epithelial morphology of organoids. **F**, TFE3 break-apart FISH assay indicated the cells with split-signals accounted for 69.5% in derived organoids, which was highly conserved with original tissue. **G**, RNA-seq indicated there was t (X; 1) (p11.2; q21) resulting in PRCC-TFE3 gene fusion in the organoid by using RNA-seq based on the gene fusion panel (GenomiCare Biotechnology, Shanghai Inc., China)

After obtaining freshly resected tumor tissue, we successfully established patient-derived tRCC organoids (Fig. 2A and B). The established tRCC organoids were propagated for three or more passages and cryopreserved. H&E data showed that both organoids and original tissue displayed a cytoplasm papillary structure harboring multiple lumen epithelium folds and invaginations (Fig. 2C). These data demonstrated the association between the morphological structure of tRCC tumors and their derived organoids. IHC staining of Ki-67 (Fig. 2D) and E-cadherin (Fig. 2E) indicated that organoids retained epithelial origin and proliferation activity.

TFE3 break-apart FISH is currently the gold standard for the identification of TFE3 rearrangements [13]. TFE3 break-apart FISH of the derived organoids indicated that the cells with split signals accounted for 69.5% of the signals (Fig. 2F). Furthermore, RNA-seq was performed, and a fusion of PRCC (exon 1, end with protein IAAPELHKGD) and TFE3 (exon 6, starting with protein CLCQGICLMC) was detected with a frequency of 15% (Fig. 2G).

Our results indicated that the tissue architecture, phenotype, cellular composition, and typical PRCC-TFE3 gene fusion of the primary tumors were reliably conserved in PRCC-TFE3 fusion organoid models.

### Development of a high-throughput phenotypical assay with PRCC-TFE3 fusion organoids

**Fig. 3:**
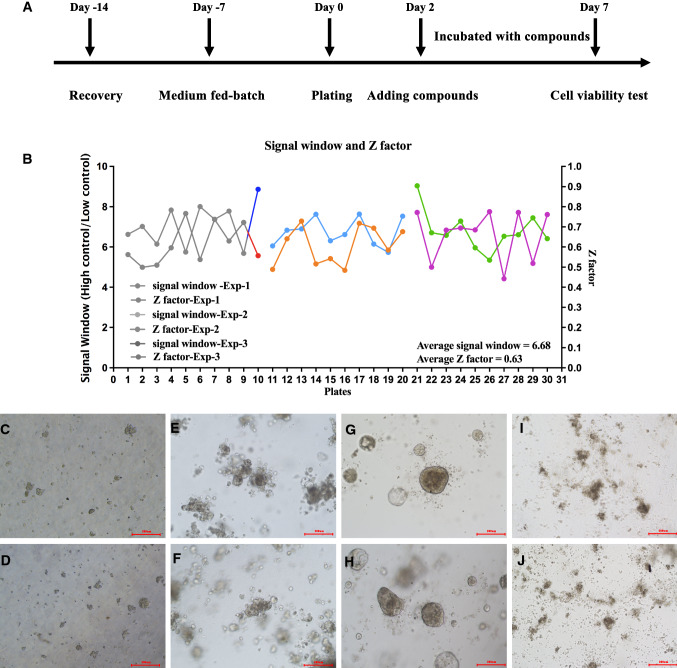
PRCC-TFE3 fusion tRCC organoids allow high throughput assay in vitro. **A** Time protocol of the preparation of PDOs and high-throughput drug screening. **B** Assay quality and robustness were evaluated with signal window (SW) and Z factor in three independent experiments of ten plates each. BEZ-235 and DMSO were employed as bottom wells and top wells, respectively. The assay showed the SW was higher than 6 and the Z factor values were between 0.44 and 0.78, which indicated the assay was qualified for high-throughput screening. **C–J**, Representative imaging of organoids at different time points, day 0 (**C** and **D**), day 2 (**E** and **F**), day 7 (**G** and **H**) of DMSO, and day 7 (**I** and **J**) of BEZ-235

After successfully establishing and characterizing PRCC-TFE3 fusion tRCC organoids, we focused on developing a three-dimensional (3D) cell-based assay that was suitable for HTS. HTS requires robust assays with high reproducibility of well-to-well and plate-to-plate assays, as well as high signal-to-noise ratios. To determine whether PRCC-TFE3 organoids could be seeded and cultured in 3D, we established a workflow employing an automated liquid-handling system (Fig. 3A).

Then, we focused on the validation of assay robustness and reproducibility by assessing plate uniformity using the parameters of SW and Z factor. Three independent experiments were conducted to evaluate the day-to-day variation. In each experiment, 10 plates were used to measure plate-to-plate variation. High control and low control wells were defined as organoids treated with medium containing DMSO and BEZ-235. Each treatment well was analyzed in quadruplicate to determine the well-to-well variation. The Z factors ranged from 0.44 to 0.78, indicating the signal-to-noise reproducibility between plates was high enough. Z factors of three independent experiments were 0.61, 0.61, and 0.66 showing the day-to-day variation was low. The average values of for SW and the Z factor were 6.88 and 0.63, respectively (Fig. 3B). These results indicated that the assay was qualified for HTS with high reproducibility. Representative images of organoids at different time points are shown in Fig. 3C–J.

### High-throughput compound library screening

To facilitate the screening of thousands of compounds across hundreds of cell lines, we used a small-molecule library containing 1,816 compounds. The target average final concentration of the compound library for screening in cell-based assays was 2 µM. Most compounds (1271, 70%) were non-oncology-related, and the remaining compounds were either chemotherapeutics or targeted oncology agents.

**Fig. 4:**
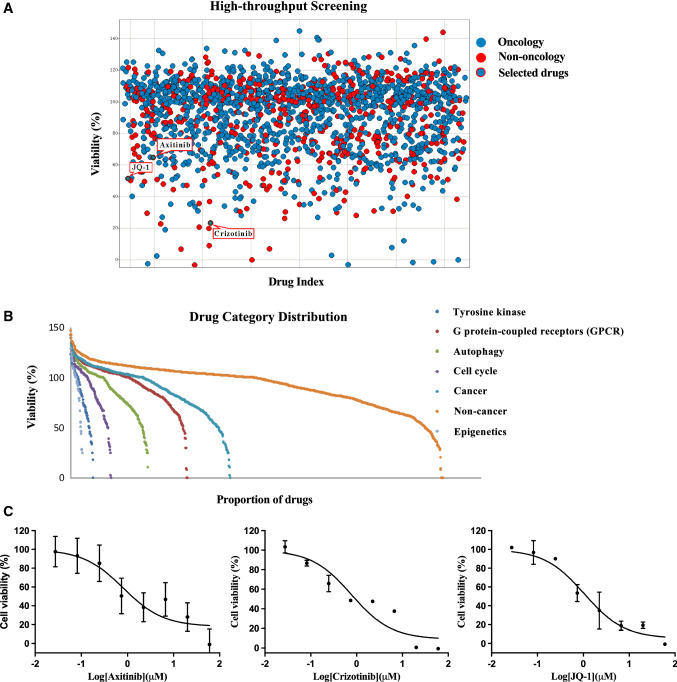
High throughput screening (HTS) with the compound library. **A** Primary screening of 1816 compounds in HTS assay. Blue spots represented the original disease was oncology, and red spots represented the original disease was non-oncology. Axitinib, crizotinib, and JQ-1 were identified. **B** Distribution of activity with compound category including tyrosine kinase, G protein-coupled receptors (GPCR), autophagy-related compounds, cell cycle-related compounds, cancer, non-cancer, and epigenetics compounds. The epigenetic, tyrosine kinase, cell cycle, and autophagy-related compounds were most frequently effective in viability assay. **C** The dose–response curves of axitinib, crizotinib, and JQ-1 in PRCC-TFE3 fusion tRCC organoids. Three selected representative leads were tested in an eight-point dose–response ranging from 60 μM to 27 nM

We designed a 2-stage screening strategy whereby the 1816 drugs were primarily screened in a single well at a single dose (2 μM). A total of 101 drugs showed more than 50% inhibition (Fig. 4A). On the other hand, when analyzing the distribution of activity with the compound category, most compounds were related to epigenetics, tyrosine kinases, the cell cycle and autophagy (Fig. 4B). Interestingly, among the 101 active drugs, most active compounds (61 out of 101, 60.4%) were originally developed for non-oncology clinical indications (Supplementary Fig s1).

As we aimed to use phenotypical drug screening to identify medicines for repurposing and assess potential mechanisms of action for TFE3 fusion tRCC, 45 chemotherapy agents with nonspecific cytotoxicity were excluded from the 101 compounds. After that, 56 compounds were retained, and axitinib was manually added to the panel. Eventually, a drug panel containing 57 compounds was designed (Supplementary Data 2) and subjected to testing via an eight-point dose–response model (doses ranging from 60 μM to 27 nM). Finally, three representative drugs, axitinib, crizotinib, and JQ-1, were selected, and the dose–response results indicated that all of them significantly inhibited cell viability (Fig. 4C).

To be noticed, axitinib is a small molecule tyrosine kinase inhibitor that inhibits multiple targets, including vascular endothelial growth factor receptor. As the PDOs did not contain vascular endothelial cells, axitinib only killed 35.4% of cell in PRCC-TFE3 fusion tRCC organoids. Axitinib activity in RCC patients has been studied in various settings, particularly as a second-line targeted treatment. Although axitinib did not reach 50% inhibition, we also included it for further investigation.

As TFE3 factors in tRCC can regulate autophagy, we then focused on autophagy-related drugs. Autophagy is required for crizotinib-induced apoptosis in MET-amplified gastric cancer cells [23]. JQ-1 is a BRD4 inhibitor, and BRD4 is a transcriptional repressor of autophagy and lysosomal function [24]. Therefore, axitinib, crizotinib, and JQ-1 were selected for further investigation.

### Cellular mechanism of inhibition of the representative drugs

**Fig. 5:**
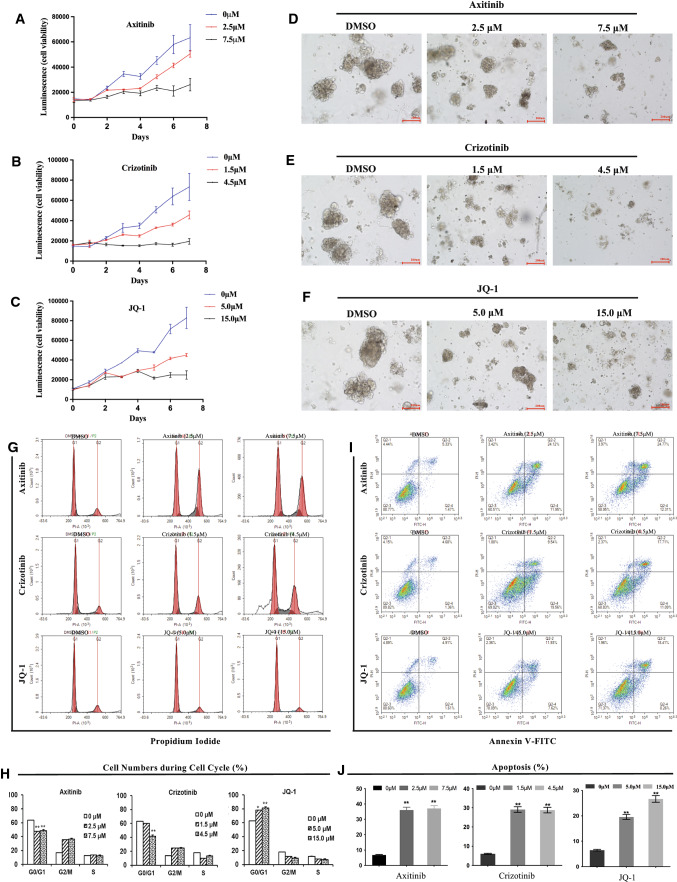
Cellular mechanism analyses of axitinib, crizotinib, and JQ-1 in PRCC-TFE3 fusion tRCC PDOs. **A–C**, axitinib, crizotinib, and JQ-1 dose-dependently suppressed the cell growth in a 7-day proliferation assay. **D–F** bright-field images (200 μm) of tRCC PDOs after incubation of 120 h with DMSO, axitinib, crizotinib, and JQ-1. Compared with the vehicle control (DMSO), three drugs of low-dose or high-dose induced the cell apoptosis significantly. **G** fluorescence activating cell sorter (FACS) showed that axitinib, crizotinib, and JQ-1 induced changes in cell counts during the cell cycle. **H** after quantitative analyses (One-way Anova) by NovoExpress v1.3.4, the percentage of cell numbers during cell cycles show that JQ-1 induced G0/G1 phase arrest, while axitinib and crizotinib induced G2/M phase arrest. **I** all three drugs could promote cell apoptosis in dose-dependent manner. Cells were treated with DMSO, axitinib (2.5 μM, 7.5 μM), crizotinib (1.5 μM, 4.5 μM), and JQ-1 (5 μM, 15 μM) for 48 h and subsequently stained with annexin v and propidium Iodide for detecting the early stage and late stage of apoptosis. **J** the quantitative data of the percentage of apoptosis cells induced by axitinib, crizotinib, and JQ-1

To further explore the cellular mechanism of axitinib, crizotinib, and JQ-1 in PDOs, we investigated the cell cycle and apoptosis effects. Proliferation assays demonstrated that all three drugs could inhibit cell proliferation in dose-dependent and time-dependent manners (Fig. 5A–C). The bright-field images (200 μm) of tRCC PDOs after incubation of 120 h with DMSO, axitinib, crizotinib, and JQ-1 showed that compared with the vehicle control (DMSO), three drugs of low-dose or high-dose induced the cell apoptosis significantly (Fig. 5D–F). Fluorescence-activated cell sorting (FACS) showed that axitinib, crizotinib, and JQ-1 induced changes in cell counts during the cell cycle (Fig. 5G). After quantitative analyses, the percentage of cells during the cell cycle showed that JQ-1 induced G0/G1 phase arrest, while axitinib and crizotinib induced G2/M phase arrest (Fig. 5H). All three drugs promoted cell apoptosis, especially early apoptosis, in a dose-dependent manner (Fig. 5I), and the cell apoptosis rates with these three drugs were shown in Fig. 5J.

### Expression profiling after drug treatment

**Fig. 6:**
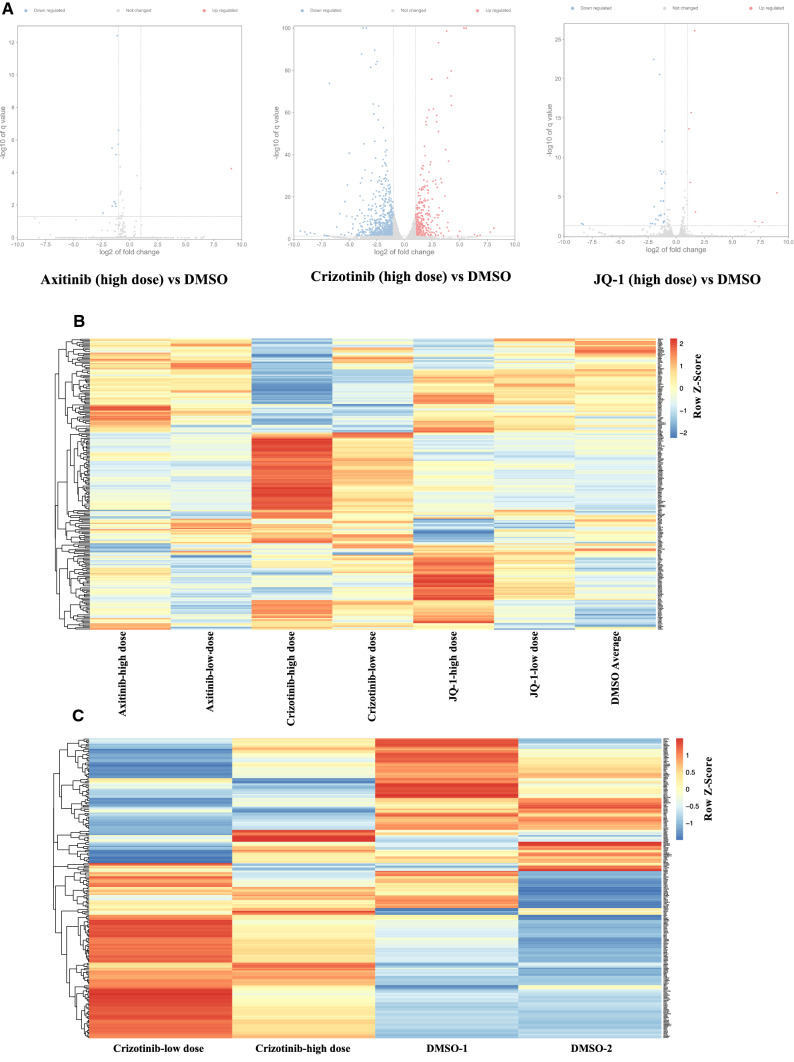
Gene profiling after compound treatments. **A** Identification of differentially expressed genes associated with axitinib, crizotinib, and JQ-1 by volcano plots. The cutoff values, log2 of fold change > 1 and *q* < 0.05, were utilized to identify differentially expressed genes. Non-changed genes were shown in grey color. Red color was indicative of up-regulated genes and blue was indicative of down-regulated genes. **B** and **C**, Analyses of expression profiles from RNA-seq data. After compound treatments, expression profiling analysis with RNA-seq indicated that autophagy-related genes (ARGs) were frequently affected by treatment of crizotinib or JQ-1 (**B**), especially for crizotinib (**C**)

To explore the underlying molecular mechanism of the antitumor effect of axitinib, crizotinib, and JQ-1, we performed whole-genome mRNA expression profiling. Total RNA isolated from PDOs treated with axitinib, crizotinib, and JQ-1 was analyzed by 3’ mRNA-Seq and compared to that from PDOs treated with DMSO. Using a log2 of fold change cutoff of 1 and a q-value cutoff of 0.05, we identified differentially expressed genes. In the axitinib high-dose group, only ten genes were upregulated, and one gene was downregulated. In the JQ-1 high-dose group, eight genes were upregulated, and 28 genes were downregulated. In the crizotinib high-dose group, 364 genes were upregulated, and 671 genes were downregulated (*q* < 0.05, log2 of fold change > 1) (Fig. 6A) (Supplementary Data 3).

We observed that ARGs were modulated by JQ-1 and crizotinib but not by axitinib (Fig. 6B). In particular, crizotinib treatment showed significant effects on ARGs (Fig. 6C) (Supplementary Data 4).

## Discussion

TFE3 fusion tRCC is an aggressive variant when present in young adults. Metastatic tRCC has a poor prognosis, and there are only a few case reports or observational studies on systemic therapy for metastatic tRCC, but the efficacy is neither representative nor ideal [8, 25]. Effective drugs are urgently needed to improve survival. PDOs are promising disease models not only for understanding disease biology but also for exploring drug efficacy in vitro before moving to animal models. The drug screening value of PDOs has been reported in liver cancer, ovarian cancer, and Crohn’s disease [26, 27]. In a recent analysis of drug responses in patients, organoid cultures were matched with the patient response up to 90% [28]. However, there are still no set-up of metastatic tRCC and patient-specific drug screening models. As TFE3 fusion tRCC has unique characteristics of transcription factor translocation, which may become a potential drug target, it is meaningful to explore promising therapeutic drugs for metastatic tRCC by employing PDOs. In our study, we established and characterized the common fusion type of metastatic tRCC PDOs for the first time and successfully performed HTS with 1816 compounds. Crizotinib and JQ-1 had promising curative value in vitro and induced significant gene alterations related to the potential pathogenesis of tRCC. These results reveal novel opportunities for using PDOs for therapeutic drug discovery.

A unique and important feature of PDOs is that they could maintain the mutational landscape of the original tumor tissue even after long-term expansion in culture and upon transplantation into mice for further research. The reproduction of original tumor genetic aberrations in a culture setting makes PDOs a potentially valuable resource in screening drug sensitivity/resistance, identifying novel therapeutics as part of a personalized medicine approach. We successfully established and cultured PDOs of metastatic tRCC with PRCC-TFE3 fusion. Thereafter, we confirmed the close relationship of the PDOs and the tumor tissue of origin by H&E, IHC, FISH, and RNA-seq assays. The complex structural histology preserved and the presence of TFE3 fusion was validated.

The prognosis of metastatic tRCC in young patients is poor, and currently available targeted therapies do not have great efficacy. In the context of exploring new effective drugs, PDOs are a promising model in the next generation of HTS studies with greater physiological relevance; these studies could involve human species specificity, regenerative applications, and modelling complex phenotypes, but the complexity of organoid cultures poses a significant challenge for miniaturization and automation [29]. Previous organoid culture studies were limited to lineages with robust, self-renewing stem cells, such as intestinal crypt cells or mammary tumors. In our study, we established a scalable, HTS-compatible assay for automated generation, maintenance, and optical analysis in standard 96-well plates. In combination with careful evaluation of reproducibility based on well-to-well and plate-to plate assays, these techniques could enable proper interpretation of library-scale drug discovery experiments. To our knowledge, a high-throughput phenotypical assay with tRCC organoids has not previously been generated automatically. Although we focused on PRCC-TFE3 fusion-affected tRCC organoids as a representative lineage, it is likely that the same general techniques could be adapted to produce other fusion types in the future.

In the present study, we found that axitinib, crizotinib, and JQ-1 arrested the cell cycle and induced the apoptosis of PRCC-TFE3 fusion tRCC organoids. Axitinib, a small molecule tyrosine kinase inhibitor, has been adopted as a second-line therapy for clear cell RCC. In addition, a multicenter phase II study with 40 non-clear cell RCCs, including 7 cases of tRCC, indicated that axitinib showed promising efficacy in terms of overall response rate (ORR) and progression-free survival (PFS) as second-line treatment in recurrent or metastatic non-clear cell RCC [30]. Therefore, we also included it for further investigation. Although it did not reach 50% inhibition during HTS, axitinib indeed induced cell cycle arrest and cell apoptosis, which indicated that it might directly kill tumor cells via other mechanisms. Crizotinib is an ATP-competitive small-molecule inhibitor of the receptor tyrosine kinases C-Met, ALK and ROS1. It inhibited cell growth and induced apoptosis in human gastric carcinoma cell lines in vitro [31]. JQ-1 displays antitumor effects in testicular germ cell tumors, leukemia, and prostate cancer by inhibiting BRD4, and BRD4 is a mediator of transcriptional elongation in the M/G1 cell cycle transition [32]. In our study, we confirmed that axitinib, crizotinib, and JQ-1 indeed promoted cell cycle arrest and apoptosis in a dose-dependent manner.

As the activity of the promoters of TFE3 fusion genes increases, overexpression of fusion proteins potentiates the intrinsic oncogenic features of TFE transcription factors [3]. TFE factors in tRCC can regulate autophagy, lysosomal biogenesis and signaling, which are considerably involved in oncogenic signaling [5]. Furthermore, TFE3 could promote the endolysosomal deprivation reaction by inducing the expression of autolysosomal genes [33, 34]. Recently, Zeng et al. demonstrated that lysosomal and autophagy pathways were significantly upregulated in TFE3-tRCC [35]. Previous studies indicated that elevated doses of crizotinib can induce autophagy in alveolar rhabdomyosarcoma cells [36] and that autophagy is required for crizotinib-induced apoptosis in MET-amplified gastric cancer cells [23]. In fact, the autophagy pathway plays a dual role in tumorigenesis. Recently, Estelle et al. indicated that autophagy has a dual role in cancer development and therapy. It could exert either prosurvival or prodeath functions in anaplastic large cell lymphoma. Differences in autophagy activity lead to differences in treatment efficacy in cancers [37]. In our RNA-seq analysis with ARGs, crizotinib was found to play a role in the antitumor effect in PRCC-TFE3 fusion tRCC organoids. We confirmed the therapeutic value of crizotinib on the PRCC-TFE3 fusion tRCC in vitro. However, the autophagy-related anticancer mechanisms of crizotinib deserve to be explored, and it was promising to further validate crizotinib in animal experiments.

Some limitations warrant discussion. tRCC has several fusion types, and our established PDOs and related drug screening results only represented the PRCC-TFE3 type. However, the PRCC-TFE3 fusion type is one of most common fusion types in tRCC. In addition, the tRCC PDO protocol has been sufficiently developed and can be applied to culture other fusion types in the future. Another limitation is the small number of tRCC PDOs; however, the first PDOs of tRCC might have a certain representative significance.

In conclusion, the organoids derived from metastatic tRCC with PRCC-TFE3 fusion that we presented here fulfilled all the criteria of a reliable in vitro cancer model, recapitulating the dominating features of the most common fusion subtype of tRCC, from histological architecture to genetic traits. The established HTS assay made drug screening amenable. Crizotinib was found to have promising antitumor activity on metastatic tRCC with PRCC-TFE3 fusion in vitro. Such organoids and HTS assays may represent a promising model system for its use both in vitro and in vivo, with a broad range of potential applications in basic and translational research assisting in the development of clinical strategies.

## Supplementary Information

Below is the link to the electronic supplementary material.

Supplementary file1 Supplementary Fig s1. Drug distribution according to different mechanisms of 101 drugs related to cell viability inhibitions. 1A, category distribution of cancer drugs. 1B, category distribution of non-oncology drugs (PDF 32 KB)

Supplementary file2 (PDF 157 KB)

Supplementary file3 (PDF 45 KB)

Supplementary file4 (PDF 109 KB)

Supplementary file5 (PDF 95 KB)

## Data Availability

The data in the current study are available from the corresponding authors on reasonable request.
