# Supplementary figures and images for: Phenotypical screening on metastatic PRCC-TFE3 fusion translocation renal cell carcinoma organoids reveals potential therapeutic agents
Source: Clin Transl Oncol. 2022 Feb 3;24(7):1333–46. doi: 10.1007/s12094-021-02774-8 (PMC9192364; doi:10.1007/s12094-021-02774-8)

**A**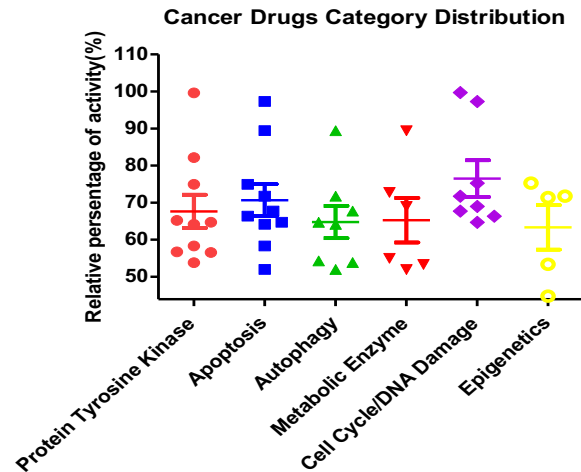**B**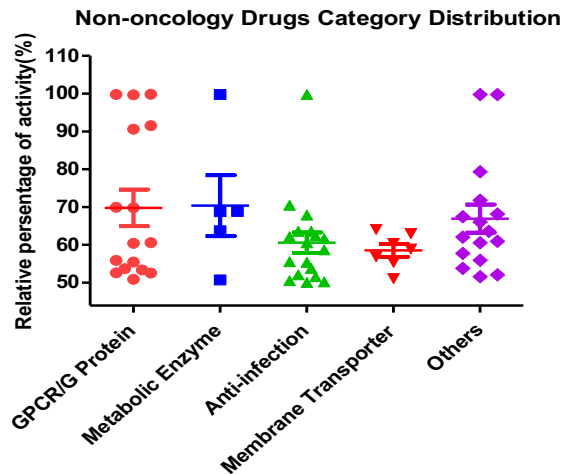

Supplement: Supplementary file 1 — Supplementary file1 Supplementary Fig s1. Drug distribution according to different mechanisms of 101 drugs related to cell viability inhibitions. 1A, category distribution of cancer drugs. 1B, category distribution of non-oncology drugs (PDF 32 KB) [file 12094_2021_2774_MOESM1_ESM.pdf]
