# Supplementary material for: Phenotypical screening on metastatic PRCC-TFE3 fusion translocation renal cell carcinoma organoids reveals potential therapeutic agents
Source: Clin Transl Oncol. 2022 Feb 3;24(7):1333–46. doi: 10.1007/s12094-021-02774-8 (PMC9192364; doi:10.1007/s12094-021-02774-8)
Supplement: Supplementary file 2 — Supplementary file2 (PDF 157 KB) [file 12094_2021_2774_MOESM2_ESM.pdf]

Supplementary Data 1. The list of 1816 compounds

No	Product Name	No	Product Name	No	Product Name	No	Product Name
1	(-)-Huperzine A	501	Desoximetasone	1001	Losartan (potassium)	1501	Selexipag
2	(-)-Menthol	502	Desvenlafaxine	1002	Loteprednol Etabonate	1502	Selumetinib
3	(-)-Sparteine (sulfate pentahydrate)	503	Desvenlafaxine (succinate hydrate)	1003	Lovastatin	1503	Seratrodast
4	(+)-Ketoconazole	504	Detomidine (hydrochloride)	1004	Loxapine	1504	Sertaconazole (nitrate)
5	(+)-α-Tocopherol	505	Dexamethasone	1005	Loxapine (succinate)	1505	Sertindole
6	(±)-Bisoprolol (hemifumarate)	506	Dexamethasone (acetate)	1006	Loxoprofen	1506	Sertraline (hydrochloride)
7	(R)-(-)-Phenylephrine (hydrochloride)	507	Dexamethasone phosphate disodium	1007	L-SelenoMethionine	1507	Setiptiline
8	(R)-Baclofen	508	Dexchlorpheniramine (maleate)	1008	L-Thyroxine	1508	Setiptiline (maleate)
9	(R)-Lansoprazole	509	Dexmedetomidine (hydrochloride)	1009	L-Thyroxine (sodium salt pentahydrate)	1509	Sildenafil
10	(S)-Timolol (Maleate)	510	Dexrazoxane hydrochloride	1010	L-Tryptophan	1510	Sildenafil (citrate)
11	(Z)-Capsaicin	511	Dextrorotation nimorazole phosphate ester	1011	Lubiprostone	1511	Silibinin
12	10-Undecenoic acid	512	Dextrose	1012	Luliconazole	1512	Silodosin
13	10-Undecenoic acid (zinc salt)	513	DHEA	1013	Lumacaftor	1513	Simeprevir
14	1-Docosanol	514	Diacerein	1014	Lumefantrine	1514	Simvastatin
15	20(S)-Ginsenoside Rg3	515	Diatrizoic acid	1015	Lusutrombopag	1515	Siponimod
16	2-Ethoxybenzamide	516	Diazoxide	1016	Macitentan	1516	Sisomicin (sulfate)
17	4-(Aminomethyl)benzoic acid	517	Dibucaine	1017	Mafenide (Acetate)	1517	Sitafloxacin (hydrate)
18	4-Aminopyridine	518	Dibucaine (hydrochloride)	1018	Malathion	1518	Sitagliptin
19	5-Aminolevulinic acid (hydrochloride)	519	Dichlorisone acetate	1019	Malotilate	1519	Sitagliptin (phosphate monohydrate)
20	5-Aminosalicylic Acid	520	Dichlorphenamide	1020	Maltitol	1520	Sivelestat
21	5-Azacytidine	521	Diclofenac	1021	Mangafodipir (trisodium)	1521	Sivelestat (sodium tetrahydrate)
22	5-Fluorouracil	522	Diclofenac (diethylamine)	1022	Manidipine (dihydrochloride)	1522	sn-Glycero-3-phosphocholine
23	6-Acetamidohexanoic acid	523	Diclofenac (Sodium)	1023	Maprotiline (hydrochloride)	1523	Sodium 4-aminosalicylate dihydrate
24	6-Aminocaproic acid	524	Dicloxacillin (Sodium hydrate)	1024	Maraviroc	1524	Sodium citrate dihydrate
25	6-Mercaptopurine hydrate	525	Didanosine	1025	Mebendazole	1525	Sodium copper chlorophyllin A
26	6-Thioguanine	526	Dienestrol	1026	Mebhydrolin	1526	Sodium diatrizoate
27	6α-Methylprednisolone 21-hemisuccinate (sodium salt)	527	Dienogest	1027	Mebhydrolin (napadisylate)	1527	Sodium Fluoride
28	9-Aminoacridine	528	Diethylcarbamazine (citrate)	1028	Meclizine (dihydrochloride)	1528	Sodium gualenate
29	Abacavir	529	Diethylstilbestrol	1029	Meclofenoxate (hydrochloride)	1529	Sodium phenylbutyrate
30	Abacavir (sulfate)	530	Diflorasone	1030	Medetomidine (hydrochloride)	1530	Sodium Picosulfate
31	Abarelix	531	Diflunisal	1031	Medroxyprogesterone (acetate)	1531	Sodium Salicylate
32	Abemaciclib (methanesulfonate)	532	Difluprednate	1032	Mefenamic acid	1532	Sodium tauroglycocholate
33	Abiraterone	533	Digitoxin	1033	Mefloquine (hydrochloride)	1533	Sofalcone
34	Abiraterone (acetate)	534	Digoxin	1034	Megestrol (Acetate)	1534	Sofosbuvir
35	Acalabrutinib	535	Dihydroergotamine (mesylate)	1035	Meglumine	1535	Solifenacin
36	Acamprosate (calcium)	536	Dihydroergotoxine (mesylate)	1036	Melatonin	1536	Solifenacin (hydrochloride)
37	Acarbose	537	Diiodohydroxyquinoline	1037	Meloxicam	1537	Solifenacin (Succinate)
38	Acebutolol (hydrochloride)	538	Diloxanide furoate	1038	Melphalan	1538	Sorafenib
39	Aceclofenac	539	Diltiazem (hydrochloride)	1039	Memantine (hydrochloride)	1539	Sorafenib (Tosylate)
40	Aceglutamide	540	Dimemorfan (phosphate)	1040	Menadione	1540	Sotalol (hydrochloride)
41	Acemetacin	541	Dimenhydrinate	1041	Menadione bisulfite (sodium)	1541	Sparfloxacin
42	Acetazolamide	542	Dimesna	1042	Menaquinone-4	1542	Spectinomycin (dihydrochloride pentahydrate)
43	Acetohexamide	543	Dimethyl fumarate	1043	Mephenesin	1543	Spectinomycin (dihydrochloride)
44	Acetohydroxamic acid	544	Dimetridazole	1044	Mepivacaine (hydrochloride)	1544	Spiramycin
45	Acetylcholine (chloride)	545	Dinoprost (tromethamine salt)	1045	Mepyramine maleate	1545	Spironolactone
46	Acetylcysteine	546	Diosmin	1046	Mequitazine	1546	Stavudine
47	Acetylleucine	547	Diphenhydramine (hydrochloride)	1047	Merbromin	1547	Stibogluconate (sodium)

48	Acetylspiramycin	548	Diphenidol (hydrochloride)	1048	Meropenem (trihydrate)	1548	Stiripentol
49	Acipimox	549	Diphenmanil (methylsulfate)	1049	Mesna	1549	Streptomycin (sulfate)
50	Acitretin	550	Diphenylpyraline (hydrochloride)	1050	Mestranol	1550	Succimer
51	Aclacinomycin A hydrochloride	551	Diphylline	1051	Metaxalone	1551	Succinylsulfathiazole
52	Aclidinium (Bromide)	552	Dipyridamole	1052	Metergoline	1552	Sucralfate
53	Acrivastine	553	Diquafosol (tetrasodium)	1053	Metformin (hydrochloride)	1553	Sucrose
54	Acyclovir	554	Dirithromycin	1054	Methacholine (chloride)	1554	Sugammadex (sodium)
55	Adapalene	555	Disulfiram	1055	Methacycline (hydrochloride)	1555	Sulbactam
56	Adefovir dipivoxil	556	Dithranol	1056	Methazolamide	1556	Sulbutiamine
57	Ademetionine (disulfate tosylate)	557	Dixyrazine	1057	Methicillin (sodium salt)	1557	Sulconazole (nitrate)
58	Adenine	558	DL-Arginine	1058	Methimazole	1558	Sulfabenzamide
59	Adenosine	559	DL-Glutamine	1059	Methocarbamol	1559	Sulfacarbamide
60	Adiphenine (hydrochloride)	560	D-Mannitol	1060	Methotrexate	1560	Sulfacetamide (Sodium)
61	Afatinib	561	Dobutamine (hydrochloride)	1061	Methoxsalen	1561	Sulfadiazine
62	Afatinib (dimaleate)	562	Docetaxel	1062	Methscopolamine (bromide)	1562	Sulfadoxine
63	Afloqualone	563	Docosahexaenoic Acid	1063	Methyl Salicylate	1563	Sulfaguanidine
64	Agomelatine	564	Docusate (Sodium)	1064	Methylbenactyzium Bromide	1564	Sulfalene
65	Agomelatine (hydrochloride)	565	Dofetilide	1065	Methylcobalamin	1565	Sulfamerazine
66	Ajmaline	566	Dolasetron	1066	Methylprednisolone	1566	Sulfameter
67	Albendazole	567	Dolasetron (Mesylate hydrate)	1067	Methylprednisolone succinate	1567	Sulfamethazine
68	Alcaftadine	568	Dolutegravir	1068	Methylthiouracil	1568	Sulfamethizole
69	Alectinib	569	Dolutegravir (sodium)	1069	Meticrane	1569	Sulfamethoxazole
70	Alectinib (Hydrochloride)	570	Domiphen (bromide)	1070	Metipranolol hydrochloride	1570	Sulfamonomethoxine
71	Alendronate (sodium hydrate)	571	Domperidone	1071	Metixene hydrochloride hydrate	1571	Sulfanilamide
72	Alfuzosin	572	Donepezil	1072	Metoclopramide	1572	Sulfaphenazole
73	Alibendol	573	Donepezil (Hydrochloride)	1073	Metoclopramide (hydrochloride hydrate)	1573	Sulfasalazine
74	Aliskiren	574	Doripenem (monohydrate)	1074	Metolazone	1574	Sulfathiazole (sodium)
75	Aliskiren (hemifumarate)	575	Dorzolamide (hydrochloride)	1075	Metoprolol (Succinate)	1575	Sulfisomidin
76	Allantoin	576	Doxapram (hydrochloride hydrate)	1076	Metronidazole	1576	Sulfisoxazole
77	Allopregnanolone	577	Doxazosin (mesylate)	1077	Metyrapone	1577	Sulfogaiacol
78	Allopurinol	578	Doxepin (Hydrochloride)	1078	Mexiletine (hydrochloride)	1578	Sulindac
79	Almitrine mesylate	579	Doxifluridine	1079	Mezlocillin (sodium)	1579	Sulpiride
80	Alogliptin (Benzoate)	580	Doxofylline	1080	Mianserin (hydrochloride)	1580	Sunitinib
81	Alosetron (Hydrochloride)	581	Doxorubicin (hydrochloride)	1081	Micafungin (sodium)	1581	Suplatast (Tosilate)
82	Alpelisib	582	Doxycycline (hyclate)	1082	Miconazole (nitrate)	1582	Suprofen
83	Alpha-Estradiol	583	Doxylamine (succinate)	1083	Midecamycin	1583	Tacrolimus (monohydrate)
84	Alprenolol	584	D-Panthenol	1084	Midostaurin	1584	Tadalafil
85	Alprenolol (hydrochloride)	585	D-Pantothenic acid (hemicalcium salt)	1085	Mifepristone	1585	Tafamidis
86	Altretamine	586	D-Pantothenic acid (sodium)	1086	Miglitol	1586	Tafamidis meglumine
87	Alverine (citrate)	587	Drofenine (hydrochloride)	1087	Miglustat (hydrochloride)	1587	Tafenoquine (Succinate)
88	Alvimopan (dihydrate)	588	Dronedarone	1088	Milnacipran ((1S-cis) hydrochloride)	1588	Tafluprost
89	Alvimopan (monohydrate)	589	Dronedarone (Hydrochloride)	1089	Milnacipran (hydrochloride)	1589	Talazoparib
90	Amantadine (hydrochloride)	590	Droperidol	1090	Milrinone	1590	Talc
91	Ambrisentan	591	Dropropizine	1091	Miltefosine	1591	Talipexole dihydrochloride
92	Ambroxol	592	Drospirenone	1092	Minaprine (dihydrochloride)	1592	Taltirelin
93	Amezinium (methylsulfate)	593	D-Sorbitol	1093	Minocycline (hydrochloride)	1593	Tamibarotene
94	Amifampridine	594	Duloxetine (hydrochloride)	1094	Minoxidil	1594	Tamoxifen
95	Amifostine	595	Dutasteride	1095	Mirabegron	1595	Tamoxifen (Citrate)
96	Amikacin (sulfate)	596	Duvelisib	1096	Miridesap	1596	Tandospirone
97	Amiloride (hydrochloride)	597	Dyclonine (hydrochloride)	1097	Miriplatin	1597	Tannic acid

98	Amiloride hydrochloride dihydrate	598	Dydrogesterone	1098	Mirodenafil (dihydrochloride)	1598	Tasimelteon
99	Aminohippurate (sodium)	599	Ebastine	1099	Mirtazapine	1599	Taurine
100	Aminophylline	600	Econazole (nitrate)	1100	Misoprostol	1600	Taurochenodeoxycholic acid
101	Amiodarone (hydrochloride)	601	Edaravone	1101	Mitiglinide (Calcium)	1601	Taurodeoxychloic Acid (sodium hydrate)
102	Amisulpride	602	Edoxaban	1102	Mitomycin C	1602	Tauroursodeoxycholate (Sodium)
103	Amitriptyline (hydrochloride)	603	Edoxaban (tosylate monohydrate)	1103	Mitotane	1603	Tavaborole
104	Amlexanox	604	Edrophonium (chloride)	1104	Mitoxantrone	1604	Tazarotene
105	Amlodipine	605	Efavirenz	1105	Mitoxantrone (dihydrochloride)	1605	Tazobactam
106	Amlodipine (besylate)	606	Efinaconazole	1106	Mizoribine	1606	Tebipenem pivoxil
107	Amlodipine (maleate)	607	Eflornithine (hydrochloride, hydrate)	1107	Moclobemide	1607	Tedizolid
108	Amodiaquin (dihydrochloride dihydrate)	608	Efonidipine (hydrochloride monoethanolate)	1108	Moexipril (hydrochloride)	1608	Tedizolid (phosphate)
109	Amorolfine (hydrochloride)	609	Eicosapentaenoic Acid	1109	Molindone (hydrochloride)	1609	Tegafur
110	Amoxapine	610	Elafibranor	1110	Molsidomine	1610	Tegaserod (maleate)
111	Amoxicillin	611	Elagolix sodium	1111	Mometasone furoate	1611	Telaprevir
112	Amoxicillin (sodium)	612	Elbasvir	1112	Monensin sodium salt	1612	Telbivudine
113	Amoxicillin (trihydrate)	613	Eletriptan (hydrobromide)	1113	Monocrotaline	1613	Telithromycin
114	Amphotericin B	614	Eliglustat	1114	Montelukast (sodium)	1614	Telmisartan
115	Ampicillin (sodium)	615	Eltrombopag	1115	Morinidazole	1615	Telotristat ethyl
116	Ampiroxicam	616	Eltrombopag (Olamine)	1116	Morinidazole (R enantiomer)	1616	Telotristat etiprate
117	Amprenavir	617	Elvitegravir	1117	Moroxydine (hydrochloride)	1617	Temocapril (hydrochloride)
118	Amsacrine	618	Embelin	1118	Mosapride (citrate)	1618	Temoporfin
119	Anagliptin	619	Emeramide	1119	Moxalactam (sodium salt)	1619	Temozolomide
120	Anagrelide (hydrochloride)	620	Emetine (dihydrochloride)	1120	Moxidectin	1620	Temsirolimus
121	Anastrozole	621	Empagliflozin	1121	Moxisylyte (hydrochloride)	1621	Teneligliptin
122	Anethole (trithione)	622	Emtricitabine	1122	Moxonidine	1622	Teneligliptin (hydrobromide)
123	Anidulafungin	623	Enalapril (maleate)	1123	Mozavaptan	1623	Teniposide
124	Aniracetam	624	Enalaprilat (dihydrate)	1124	Mupirocin	1624	Tenofovir
125	Anisindione	625	Enasidenib	1125	Mycophenolate Mofetil	1625	Tenofovir (Disoproxil Fumarate)
126	Anisodamine	626	Enasidenib (mesylate)	1126	Mycophenolic acid	1626	Tenofovir (Disoproxil)
127	Antazoline (hydrochloride)	627	Enoxacin (hydrate)	1127	Nabumetone	1627	Tenofovir (hydrate)
128	Antipyrine	628	Entacapone	1128	Nadifloxacin	1628	Tenofovir alafenamide
129	Apalutamide	629	Entecavir (monohydrate)	1129	Nafamostat (mesylate)	1629	Tenofovir alafenamide fumarate
130	Apatinib	630	Entrectinib	1130	Nafcillin (sodium monohydrate)	1630	Tenofovir alafenamide hemifumarate
131	Apixaban	631	Enzalutamide	1131	Naftidrofuryl (oxalate)	1631	Tenoxicam
132	Apremilast	632	Epalrestat	1132	Naftifine (hydrochloride)	1632	Teprenone
133	Aprepitant	633	Eperisone (Hydrochloride)	1133	Naftopidil	1633	Terazosin (hydrochloride dihydrate)
134	Apronal	634	Epinastine	1134	Nalfurafine (hydrochloride)	1634	Terbinafine
135	Aprotinin	635	Epirubicin (hydrochloride)	1135	Nalidixic acid	1635	Terbinafine hydrochloride
136	Argatroban (monohydrate)	636	Eplerenone	1136	Naloxegol (oxalate)	1636	Terbutaline (sulfate)
137	Argipressin	637	Eprosartan (mesylate)	1137	Naphazoline (hydrochloride)	1637	Terconazole
138	Aripiprazole	638	Eptifibatide	1138	Naproxen	1638	Teriflunomide
139	Artemether	639	Eravacycline (dihydrochloride)	1139	Naproxen (sodium)	1639	Teriparatide
140	Artemisinin	640	Erdafitinib	1140	Naratriptan (hydrochloride)	1640	Terlipressin
141	Artemotil	641	Erdosteine	1141	Natamycin	1641	Terpin (hydrate)
142	Arterolane	642	Erismodegib	1142	Nateglinide	1642	Tetrabenazine
143	Artesunate	643	Erismodegib diphosphate	1143	Nebivolol (hydrochloride)	1643	Tetracaine
144	Articaine (hydrochloride)	644	Erlotinib	1144	Nedaplatin	1644	Tetracycline (hydrochloride)
145	Asenapine	645	Erlotinib (Hydrochloride)	1145	Nedocromil	1645	Tetrahydrobiopterin
146	Asenapine (hydrochloride)	646	Ertapenem sodium	1146	Nefazodone (hydrochloride)	1646	Tetrahydrozoline (hydrochloride)
147	Asenapine (maleate)	647	Ertugliflozin	1147	Nefopam (hydrochloride)	1647	Tetramisole (hydrochloride)

148	Aspirin	648	Ertugliflozin L-pyroglutamic acid	1148	Nelarabine	1648	Tezacaftor
149	Asunaprevir	649	Erythromycin	1149	Nelfinavir	1649	Thalidomide
150	Ataluren	650	Erythromycin Ethylsuccinate	1150	Nelfinavir (Mesylate)	1650	Theophylline
151	Atazanavir (sulfate)	651	Esaxerenone	1151	Neomycin (sulfate)	1651	Thiabendazole
152	Atenolol	652	Escin	1152	Neostigmine (Bromide)	1652	Thiamine hydrochloride
153	Atomoxetine (hydrochloride)	653	Escitalopram (oxalate)	1153	Neostigmine (methyl sulfate)	1653	Thiamine monochloride
154	Atorvastatin (hemicalcium salt)	654	Eslicarbazepine (acetate)	1154	Nepafenac	1654	Thiamine nitrate
155	Atovaquone	655	Esmolol (hydrochloride)	1155	Neratinib	1655	Thiamphenicol
156	Atracurium (besylate)	656	Esomeprazole magnesium	1156	Netilmicin (sulfate)	1656	Thioridazine (hydrochloride)
157	Atrasentan (hydrochloride)	657	Estradiol	1157	Netupitant	1657	Thio-TEPA
158	Atropine	658	Estradiol (benzoate)	1158	Nevirapine	1658	Thonzonium (bromide)
159	Atropine (sulfate monohydrate)	659	Estradiol (cypionate)	1159	Niacin	1659	Tiagabine (hydrochloride)
160	Atropine methyl bromide	660	Estramustine (phosphate sodium)	1160	Nicardipine (Hydrochloride)	1660	Tiamulin (fumarate)
161	Auranofin	661	Estriol	1161	Nicergoline	1661	Ticagrelor
162	Avanafil	662	Estrone	1162	Niclosamide	1662	Ticarcillin (disodium)
163	Avibactam (sodium hydrate)	663	Estropipate	1163	Nicorandil	1663	Ticlopidine (hydrochloride)
164	Avibactam (sodium)	664	Etamivan	1164	Nicotinamide	1664	Tigecycline
165	Avobenzone	665	Ethacridine (lactate monohydrate)	1165	Nifedipine	1665	Tigecycline (tetramesylate)
166	Axitinib	666	Ethacridine (lactate)	1166	Niflumic acid	1666	Tilorone (dihydrochloride)
167	Azaperone	667	Ethambutol (dihydrochloride)	1167	Nifuratel	1667	Tinidazole
168	Azaphen (dihydrochloride monohydrate)	668	Ethamsylate	1168	Nifuroxazide	1668	Tinoridine hydrochloride
169	Azasetron (hydrochloride)	669	Ethionamide	1169	Nifurtimox	1669	Tioconazole
170	Azatadine (dimaleate)	670	Ethosuximide	1170	Nikethamide	1670	Tiopronin
171	Azathioprine	671	Ethoxzolamide	1171	Nilotinib	1671	Tiotropium (Bromide)
172	AZD7545	672	Ethynodiol (diacetate)	1172	Nilotinib (monohydrochloride monohydrate)	1672	Tipiracil (hydrochloride)
173	Azelaic acid	673	Ethynyl Estradiol	1173	Nilvadipine	1673	Tipranavir
174	Azelastine (hydrochloride)	674	Etidronic acid	1174	Nimesulide	1674	Tiratricol
175	Azelnidipine	675	Etodolac	1175	Nimodipine	1675	Tirofiban (hydrochloride monohydrate)
176	Azilsartan	676	Etofenamate	1176	Nimorazole	1676	Tizanidine (hydrochloride)
177	Azilsartan (medoxomil)	677	Etofylline	1177	Nintedanib	1677	Tobramycin
178	Azithromycin	678	Etomidate	1178	Nintedanib esylate	1678	Toceranib
179	Azlocillin (sodium salt)	679	Etomidate (hydrochloride)	1179	Niraparib	1679	Tocofersolan
180	Aztreonam	680	Etoposide	1180	Niraparib hydrochloride	1680	Tofacitinib
181	Bacampicillin (hydrochloride)	681	Etoricoxib	1181	Niraparib tosylate	1681	Tofacitinib (citrate)
182	Balofloxacin	682	Etravirine	1182	Nitazoxanide	1682	Tofogliflozin (hydrate)
183	Baloxavir marboxil	683	Etretinate	1183	Nitisinone	1683	Tolazamide
184	Balsalazide	684	Etripamil	1184	Nitrendipine	1684	Tolazoline (hydrochloride)
185	Bambuterol hydrochloride	685	Evans Blue	1185	Nitrofurantoin	1685	Tolbutamide
186	Baricitinib	686	Exemestane	1186	Nitrofurazone	1686	Tolcapone
187	Baricitinib (phosphate)	687	Ezetimibe	1187	Nitroprusside (disodium dihydrate)	1687	Tolfenamic Acid
188	Bazedoxifene (acetate)	688	Fabomotizole (hydrochloride)	1188	Nitroxoline	1688	Tolmetin (sodium dihydrate)
189	Bedaquiline	689	Fadrozole	1189	Nizatidine	1689	Toloxatone
190	Bedaquiline (fumarate)	690	Famciclovir	1190	Nonivamide	1690	Tolperisone (hydrochloride)
191	Bekanamycin	691	Famotidine	1191	Norepinephrine	1691	Tolterodine
192	Belinostat	692	Faropenem daloxate	1192	Norepinephrine (bitartrate monohydrate)	1692	Tolterodine (tartrate)
193	Belotecan (hydrochloride)	693	Faropenem sodium	1193	Norethindrone acetate	1693	Tolvaptan
194	Bemegride	694	Fasudil (Hydrochloride)	1194	Norfloxacin	1694	Topiramate
195	Benactyzine hydrochloride	695	Favipiravir	1195	Nortriptyline (hydrochloride)	1695	Topiroxostat
196	Benazepril (hydrochloride)	696	Febuxostat	1196	Norvancomycin (hydrochloride)	1696	Topotecan (Hydrochloride)
197	Bendamustine (hydrochloride)	697	Felbamate	1197	Noscapine	1697	Toremifene (Citrate)

198	Bendazol	698	Felbinac	1198	Novobiocin (Sodium)	1698	Torsemide
199	Benfluorex hydrochloride	699	Felodipine	1199	Nystatin	1699	Tosufloxacin (tosylate hydrate)
200	Benfotiamine	700	Felypressin	1200	Obeticholic acid	1700	Trametinib
201	Benidipine (hydrochloride)	701	Fenbufen	1201	Octenidine (dihydrochloride)	1701	Trametinib (DMSO solvate)
202	Benorilate	702	Fenofibrate	1202	Octinoxate	1702	Trandolapril
203	Benserazide hydrochloride (Synonyms: Serazide; Ro 4-4602)	703	Fenofibric acid	1203	Octocrylene	1703	Tranexamic acid
204	Bentiromide	704	Fenoldopam (mesylate)	1204	Octreotide (acetate)	1704	Tranilast
205	Benzamil hydrochloride	705	Fenoprofen (Calcium hydrate)	1205	Ofloxacin	1705	Tranylcypromine (hemisulfate)
206	Benzbromarone	706	Fenspiride (Hydrochloride)	1206	Olanzapine	1706	Trapidil
207	Benznidazol	707	Fenticonazole (Nitrate)	1207	Olaparib	1707	Travoprost
208	Benzocaine	708	Fertirelin	1208	Olmesartan	1708	Trazodone (hydrochloride)
209	Benzthiazide	709	Fesoterodine (fumarate)	1209	Olmesartan (medoxomil)	1709	Trelagliptin
210	Benztropine (mesylate)	710	Fexofenadine (hydrochloride)	1210	Olopatadine (hydrochloride)	1710	Trelagliptin (succinate)
211	Benzydamine (hydrochloride)	711	Fidaxomicin	1211	Olprinone (Hydrochloride)	1711	Treosulfan
212	Benzyl alcohol	712	Fimasartan	1212	Olsalazine (Disodium)	1712	Treprostinil (sodium)
213	Benzyl benzoate	713	Finafloxacin	1213	Omarigliptin	1713	Triamcinolone
214	Bephenium (hydroxynaphthoate)	714	Finasteride	1214	Ombitasvir	1714	Triamcinolone (acetonide)
215	Bepotastine (Beslilate)	715	Fingolimod	1215	Omeprazole	1715	Triamcinolone hexacetonide
216	Bepridil hydrochloride	716	Fingolimod (hydrochloride)	1216	Opicapone	1716	Triamterene
217	Berberine (chloride hydrate)	717	Firocoxib	1217	Orlistat	1717	Tricaprilin
218	Berberine (chloride)	718	Flavin Adenine Dinucleotide Disodium	1218	Ornidazole	1718	Trichlormethiazide
219	Besifloxacin (Hydrochloride)	719	Flavoxate (hydrochloride)	1219	Ornidazole (Levo-)	1719	Triclabendazole
220	Bestatin	720	Flecainide (acetate)	1220	Ornipressin	1720	Triclosan
221	Betahistine (dihydrochloride)	721	Flibanserin	1221	Orotic acid	1721	Trifluoperazine (dihydrochloride)
222	Betaine (hydrochloride)	722	Floxuridine	1222	Orphenadrine (citrate)	1722	Triflupromazine (hydrochloride)
223	Betamethasone	723	Flubendazole	1223	Osalmid	1723	Trifluridine
224	Betamethasone dipropionate	724	Flucloxacillin sodium	1224	Oseltamivir (acid)	1724	Triflusal
225	Betamipron	725	Fluconazole	1225	Oseltamivir (phosphate)	1725	Trihexyphenidyl (hydrochloride)
226	Betaxolol	726	Flucytosine	1226	Osimertinib	1726	Trilostane
227	Betaxolol (hydrochloride)	727	Fludarabine	1227	Osimertinib mesylate	1727	Trimebutine (maleate)
228	Bethanechol (chloride)	728	Fludarabine (phosphate)	1228	Ospemifene	1728	Trimetazidine (dihydrochloride)
229	Betrixaban	729	Fludrocortisone (acetate)	1229	Otilonium (bromide)	1729	Trimethadione
230	Bexarotene	730	Flufenamic acid	1230	Ouabain (Octahydrate)	1730	Trimethobenzamide hydrochloride
231	Bezafibrate	731	Flumazenil	1231	Oxaceprol	1731	Trimethoprim
232	Biapenem	732	Flumethasone	1232	Oxacillin (sodium monohydrate)	1732	Trimipramine (maleate)
233	Bicalutamide	733	Flunarizine (dihydrochloride)	1233	Oxaliplatin	1733	Tripelennamine (hydrochloride)
234	Bictegravir	734	Flunisolide	1234	Oxantel (pamoate)	1734	Troglitazone
235	Bicyclol	735	Fluocinolone (Acetonide)	1235	Oxaprozin	1735	Tropicamide
236	Bifonazole	736	Fluocinonide	1236	Oxcarbazepine	1736	Tropisetron
237	Bilastine	737	Fluorescein	1237	Oxeladin (citrate)	1737	Tropisetron (Hydrochloride)
238	Bimatoprost	738	Fluoxetine (hydrochloride)	1238	Oxethazaine	1738	Trospium (chloride)
239	Binimetinib	739	Fluphenazine (dihydrochloride)	1239	Oxiconazole nitrate	1739	Troxipide
240	Biotin	740	Flupirtine (Maleate)	1240	Oxiracetam	1740	Tucidinostat
241	Biperiden (Hydrochloride)	741	Fluralaner	1241	Oxolamine (citrate)	1741	Tulobuterol (hydrochloride)
242	Bisacodyl	742	Flurbiprofen	1242	Oxybenzone	1742	Udenafil
243	Bisoctrizole	743	Flutamide	1243	Oxybutynin	1743	Ulipristal (acetate)
244	Bivalirudin (TFA)	744	Fluticasone (propionate)	1244	Oxybutynin (chloride)	1744	Umeclidinium (bromide)
245	Bleomycin (sulfate)	745	Fluvastatin (sodium)	1245	Oxyclozanide	1745	Upadacitinib
246	Blonanserin	746	Fluvoxamine (maleate)	1246	Oxytetracycline	1746	Urapidil
247	Boceprevir	747	Folic acid	1247	Oxytocin (acetate)	1747	Urea

248	Bortezomib	748	Fomepizole	1248	Ozagrel	1748	Uridin
249	Bosentan	749	Fondaparinux (sodium)	1249	Ozagrel sodium	1749	Ursodiol
250	Bosentan (hydrate)	750	Forodesine (hydrochloride)	1250	Ozenoxacin	1750	Vadadustat
251	Bosutinib	751	Fosamprenavir	1251	Paclitaxel	1751	VAL-083
252	Bremelanotide (Acetate)	752	Fosfluconazole	1252	Palbociclib (hydrochloride)	1752	Valacyclovir (hydrochloride)
253	Brexpiprazole	753	Fosfomycin (calcium)	1253	Palbociclib (isethionate)	1753	Valbenazine
254	Brimonidine	754	Fosinopril (sodium)	1254	Paliperidone	1754	Valdecoxib
255	Brimonidine (tartrate)	755	Fosphenytoin (disodium)	1255	Palonosetron (Hydrochloride)	1755	Valganciclovir (hydrochloride)
256	Brinzolamide	756	Fostamatinib Disodium	1256	Pamidronate (disodium pentahydrate)	1756	Valnemulin (Hydrochloride)
257	Brivudine	757	Framycetin	1257	Pamidronic acid	1757	Valproic acid
258	Bromfenac (sodium hydrate)	758	Fructose	1258	Pancuronium (dibromide)	1758	Valproic acid (sodium salt)
259	Bromhexine (hydrochloride)	759	Fudosteine	1259	Panobinostat	1759	Valpromide
260	Bromisoval	760	Fulvestrant	1260	Pantoprazole (sodium)	1760	Valrubicin
261	Bromocriptine (mesylate)	761	Fumaric acid	1261	Parecoxib	1761	Valsartan
262	Brompheniramine (maleate)	762	Furagin	1262	Parecoxib (Sodium)	1762	Vancomycin (hydrochloride)
263	Bronopol	763	Furazolidone	1263	Pargyline (hydrochloride)	1763	Vandetanib
264	Broxyquinoline	764	Furosemide	1264	Paritaprevir	1764	Vardenafil (hydrochloride)
265	Bucladesine (calcium salt)	765	Furosemide (sodium)	1265	Paromomycin (sulfate)	1765	Varenicline
266	Bucladesine (sodium salt)	766	Fursultiamine	1266	Paroxetine (hydrochloride)	1766	Varenicline (Hydrochloride)
267	Budesonide	767	Fusidic acid (sodium salt)	1267	Pasiniazid	1767	Varenicline (Tartrate)
268	Bufexamac	768	Gabapentin	1268	Pasireotide (ditrifluoroacetate)	1768	Vecuronium (bromide)
269	Buflomedil (hydrochloride)	769	Gabapentin (hydrochloride)	1269	Pazopanib	1769	Velpatasvir
270	Bumetanide	770	Gabapentin enacarbil	1270	Pazopanib (Hydrochloride)	1770	Vemurafenib
271	Buspirone (hydrochloride)	771	Gabexate (mesylate)	1271	Pazufloxacin (mesylate)	1771	Venetoclax
272	Busulfan	772	Gadobutrol	1272	Peficitinib	1772	Venlafaxine (hydrochloride)
273	Butamben	773	Gadodiamide	1273	Pefloxacin (mesylate)	1773	Verapamil (hydrochloride)
274	Butenafine (Hydrochloride)	774	Gadodiamide (hydrate)	1274	Pemetrexed	1774	Vernakalant (Hydrochloride)
275	Butoconazole (nitrate)	775	Gadoxetate (Disodium)	1275	Pemetrexed (disodium hemipenta hydrate)	1775	Verteporfin
276	Butylphthalide	776	Galanthamine	1276	Pemetrexed (disodium)	1776	Vesnarinone
277	Cabazitaxel	777	Galanthamine (hydrobromide)	1277	Pemirolast (potassium)	1777	Vidarabine
278	Cabergoline	778	Gallamine Triethiodide	1278	Penbutolol (sulfate)	1778	Vigabatrin
279	Cabozantinib	779	Gallic acid	1279	Penciclovir	1779	Vigabatrin (Hydrochloride)
280	Cabozantinib (S-malate)	780	Gamithromycin	1280	Penfluridol	1780	Vilanterol (trifenatate)
281	Caffeic acid	781	Ganciclovir	1281	Penicillamine	1781	Vilazodone
282	Calcium dobesilate	782	Garenoxacin (Mesylate hydrate)	1282	Pentamidine (isethionate)	1782	Vilazodone (Hydrochloride)
283	Camostat (mesylate)	783	Gastrodenol	1283	Pentostatin	1783	Vildagliptin
284	Camphor	784	Gastrodin	1284	Pentoxifylline	1784	Vinblastine (sulfate)
285	Canagliflozin	785	Gatifloxacin	1285	Peramivir (trihydrate)	1785	Vincamine
286	Canagliflozin (hemihydrate)	786	Gefarnate	1286	Pergolide (mesylate)	1786	Vinorelbine (ditartrate)
287	Candesartan	787	Gefitinib	1287	Perhexiline maleate	1787	Vinpocetine
288	Candesartan (Cilexetil)	788	Gefitinib (hydrochloride)	1288	Perindopril (erbumine)	1788	Vismodegib
289	Canrenone	789	Gemcitabine	1289	Permethrin	1789	Vitamin B12
290	Capecitabine	790	Gemcitabine (hydrochloride)	1290	Perphenazine	1790	Vitamin D2
291	Capreomycin (sulfate)	791	Gemfibrozil	1291	Pexidartinib	1791	Vitamin K1
292	Capsaicin	792	Gemifloxacin (mesylate)	1292	Pexidartinib (hydrochloride)	1792	Voglibose
293	Captopril	793	Gestodene	1293	Phenazopyridine (hydrochloride)	1793	Vonoprazan
294	Carbamazepine	794	Gestrinone	1294	Phenelzine (sulfate)	1794	Vonoprazan (Fumarate)
295	Carbamoylcholine (chloride)	795	Gimeracil	1295	Phenindione	1795	Vorapaxar
296	Carbazochrome (sodium sulfonate)	796	Glafenine (hydrochloride)	1296	Pheniramine (Maleate)	1796	Voriconazole
297	Carbetapentane (citrate)	797	Glasdegib	1297	Phenoxybenzamine (hydrochloride)	1797	Vorinostat

298	Carbetocin	798	Glecaprevir	1298	Phenprocoumon	1798	Vortioxetine
299	Carbidopa	799	Glibenclamide	1299	Phentolamine (mesylate)	1799	Vortioxetine (hydrobromide)
300	Carbimazole	800	Gliclazide	1300	Phenylbutazone	1800	Warfarin
301	Carbinoxamine maleate salt	801	Glimepiride	1301	Phenytoin	1801	Xylitol
302	Carboxin	802	Glipizide	1302	Phenytoin (sodium)	1802	Xylometazoline (hydrochloride)
303	Carfilzomib	803	Gliquidone	1303	Phthalylsulfacetamide	1803	Yohimbine (Hydrochloride)
304	Carglumic Acid	804	Gluconate (Calcium)	1304	Pidotimod	1804	Zafirlukast
305	Cariprazine	805	Gluconate (sodium)	1305	Pimavanserin	1805	Zalcitabine
306	Cariprazine (hydrochloride)	806	Glucosamine (hydrochloride)	1306	Pimavanserin tartrate	1806	Zaltoprofen
307	Carmofur	807	Glycerol phenylbutyrate	1307	Pimecrolimus	1807	Zanamivir
308	Carmustine	808	Glycine	1308	Pimozide	1808	Zidovudine
309	Carprofen	809	Glycopyrrolate	1309	Pinaverium bromide	1809	Zileuton
310	Carteolol hydrochloride	810	Gonadorelin (acetate)	1310	Pindolol	1810	Ziprasidone
311	Carvedilol	811	Goserelin (acetate)	1311	Pioglitazone	1811	Ziprasidone (hydrochloride monohydrate)
312	Caspofungin (Acetate)	812	Gramicidin	1312	Pioglitazone (hydrochloride)	1812	Zofenopril (calcium)
313	Catechin	813	Granisetron (Hydrochloride)	1313	Pipemidic acid	1813	Zoledronic acid (monohydrate)
314	Cefaclor	814	Grazoprevir	1314	Piperacillin (sodium)	1814	Zonisamide
315	Cefadroxil	815	Grazoprevir potassium salt	1315	Piperidolate	1815	α-Lipoic Acid
316	Cefamandole (nafate)	816	Griseofulvin	1316	Piperidolate (hydrochloride)	1816	β-Carotene
317	Cefazolin (sodium)	817	Guacetisal	1317	Piperonyl butoxide		
318	Cefdinir	818	Guaiacol	1318	Pipobroman		
319	Cefditoren (Pivoxil)	819	Guaifenesin	1319	Piracetam		
320	Cefepime (Dihydrochloride Monohydrate)	820	Guanabenz (Acetate)	1320	Pirarubicin (Hydrochloride)		
321	Cefixime	821	Guanethidine (sulfate)	1321	Pirenzepine (dihydrochloride)		
322	Cefmenoxime (hydrochloride)	822	Guanfacine (hydrochloride)	1322	Pirfenidone		
323	Cefmetazole (sodium)	823	Guanidine (hydrochloride)	1323	Piribedil		
324	Cefonicid (sodium)	824	Halcinonide	1324	Pirmenol (hydrochloride)		
325	Cefoperazone	825	Halobetasol (propionate)	1325	Piroctone olamine		
326	Cefoperazone (sodium salt)	826	Haloperidol	1326	Piromidic acid		
327	Cefoselis (sulfate)	827	Helicid	1327	Piroxicam		
328	Cefotaxime (sodium salt)	828	Heparin (sodium salt)	1328	Pitavastatin (Calcium)		
329	Cefoxitin (sodium)	829	Heptaminol (hydrochloride)	1329	Pitolisant (hydrochloride)		
330	Cefozopran (hydrochloride)	830	Hexachlorophene	1330	Pivmecillinam (hydrochloride)		
331	Cefprozil (monohydrate)	831	Hexaminolevulinate (hydrochloride)	1331	Pixantrone (dimaleate)		
332	Cefsulodin (sodium)	832	Hexylresorcinol	1332	Pizotifen		
333	Ceftaroline fosamil	833	Histamine	1333	Plerixafor		
334	Ceftazidime	834	Histamine (phosphate)	1334	Plerixafor (octahydrochloride)		
335	Ceftibuten (dihydrate)	835	Homatropine (Bromide)	1335	Podofilox		
336	Ceftizoxime	836	Homatropine (methylbromide)	1336	Pomalidomide		
337	Ceftriaxone (sodium salt)	837	Homoharringtonine	1337	Ponatinib		
338	Cefuroxime (sodium)	838	Hydralazine (hydrochloride)	1338	Posaconazole		
339	Celecoxib	839	Hydrocortisone	1339	Pralatrexate		
340	Cephalexin	840	Hydrocortisone (acetate)	1340	Pralidoxime (chloride)		
341	Cephalexin (monohydrate)	841	Hydrocortisone 17-butyrate	1341	Pramipexole (dihydrochloride)		
342	Cephalothin (sodium)	842	Hydrocortisone buteprate	1342	Pramiracetam		
343	Cephradine	843	Hydrocortisone cypionate	1343	Pramocaine (hydrochloride)		
344	Ceritinib	844	Hydroquinidine	1344	Pranlukast		
345	Ceritinib dihydrochloride	845	Hydroxychloroquine sulfate	1345	Pranlukast (hemihydrate)		
346	Cetilistat	846	Hydroxyfasudil	1346	Pranoprofen		
347	Cetirizine (dihydrochloride)	847	Hydroxyfasudil (hydrochloride)	1347	Prasugrel		

348	Cetylpyridinium (chloride monohydrate)	848	Hydroxyprogesterone caproate	1348	Prasugrel (hydrochloride)		
349	Cevimeline (hydrochloride hemihydrate)	849	Hydroxyurea	1349	Pravastatin (sodium)		
350	Cevimeline (hydrochloride)	850	Hydroxyzine (dihydrochloride)	1350	Praziquantel		
351	Chenodeoxycholic Acid	851	Ibandronate (Sodium Monohydrate)	1351	Prazosin (hydrochloride)		
352	Chlorambucil	852	Ibrutinib	1352	Prednisolone		
353	Chlorcyclizine (hydrochloride)	853	Ibrutinib Racemate	1353	Prednisolone (21-acetate)		
354	Chlorhexidine	854	Ibudilast	1354	Prednisolone (disodium phosphate)		
355	Chlorhexidine (digluconate)	855	Ibuprofen	1355	Prednisone		
356	Chlormadinone (acetate)	856	Ibuprofen piconol	1356	Prednisone acetate		
357	Chlormethine (hydrochloride)	857	Ibutilide (fumarate)	1357	Pretomanid		
358	Chlormezanone	858	Icatibant	1358	Prilocaine		
359	Chlorocresol	859	Icotinib	1359	Primaquine (Diphosphate)		
360	Chloroquine (diphosphate)	860	Icotinib (Hydrochloride)	1360	Primidone		
361	Chlorothiazide	861	Idarubicin (hydrochloride)	1361	Probenecid		
362	Chloroxine	862	Idebenone	1362	Probucol		
363	Chlorpheniramine (maleate)	863	Idelalisib	1363	Procainamide (hydrochloride)		
364	Chlorphenoxamine	864	Idoxuridine	1364	Procarbazine (Hydrochloride)		
365	Chlorpropamide	865	Idramantone	1365	Procyclidine (hydrochloride)		
366	Chlorprothixene	866	Ifenprodil (tartrate)	1366	Progesterone		
367	Chlorquinaldol	867	Iguratimod	1367	Proglumide		
368	Chlortetracycline (hydrochloride)	868	i-Inositol	1368	Proguanil		
369	Chlorthalidone	869	Ilaprazole	1369	Promazine (hydrochloride)		
370	Chlorzoxazone	870	Iloperidone	1370	Propafenone (hydrochloride)		
371	Cholic acid	871	Iloprost	1371	Propantheline (bromide)		
372	Choline (chloride)	872	Imatinib	1372	Proparacaine (Hydrochloride)		
373	Choline Fenofibrate	873	Imatinib (Mesylate)	1373	Propoxycaine (hydrochloride)		
374	Chromocarb	874	Imidafenacin	1374	Propranolol (hydrochloride)		
375	Ciclesonide	875	Imidapril (hydrochloride)	1375	Propylthiouracil		
376	Ciclopirox	876	Imipramine (hydrochloride)	1376	Propyphenazone		
377	Ciclopirox (olamine)	877	Imiquimod	1377	Prostaglandin E2		
378	Cidofovir	878	Imrecoxib	1378	Prothionamide		
379	Cilastatin	879	Indacaterol (maleate)	1379	Protoporphyrin IX		
380	Cilazapril (monohydrate)	880	Indapamide	1380	Protriptyline (hydrochloride)		
381	Cilnidipine	881	Indinavir (sulfate)	1381	Proxyphylline		
382	Cilostazol	882	Indomethacin	1382	Prucalopride		
383	Cinacalcet	883	Ingenol	1383	Prucalopride (succinate)		
384	Cinacalcet (hydrochloride)	884	Inosine pranobex	1384	Prulifloxacin		
385	Cinepazide (Maleate)	885	Iohexol	1385	Pyrantel (pamoate)		
386	Cinnarizine	886	Iopamidol	1386	Pyrantel (tartrate)		
387	Cinobufotalin	887	Iopanoic acid	1387	Pyrazinamide		
388	Cinoxacin	888	Iopromide	1388	Pyridostigmine (bromide)		
389	Ciprofibrate	889	Ioversol	1389	Pyridoxine (hydrochloride)		
390	Cisapride	890	Ipratropium (bromide)	1390	Pyrimethamine		
391	Cisatracurium (besylate)	891	Iproniazid (phosphate)	1391	Pyrithioxin (dihydrochloride)		
392	Citalopram (hydrobromide)	892	Irbesartan	1392	Pyrvinium pamoate		
393	Citicoline	893	Irinotecan	1393	Quetiapine		
394	Citicoline sodium salt	894	Irinotecan (hydrochloride trihydrate)	1394	Quetiapine (fumarate)		
395	Citric acid	895	Irinotecan (hydrochloride)	1395	Quinagolide (hydrochloride)		
396	Citric acid (trilithium salt tetrahydrate)	896	Irsogladine	1396	Quinapril (hydrochloride)		
397	Cladribine	897	Isoconazole (nitrate)	1397	Quinestrol		

398	Clarithromycin	898	Isoniazid	1398	Quinidine		
399	Clebopride (malate)	899	Isosorbide	1399	Quinidine hydrochloride monohydrate		
400	Clemastine (fumarate)	900	Isosorbide mononitrate	1400	Quinine		
401	Clemizole (hydrochloride)	901	Isotretinoin	1401	Quinine (hydrochloride dihydrate)		
402	Clevidipine	902	Isradipine	1402	Rabeprazole (sodium)		
403	Clevudine	903	Istradefylline	1403	Racecadotril		
404	Clindamycin (hydrochloride)	904	Itopride (hydrochloride)	1404	Raloxifene (hydrochloride)		
405	Clindamycin (phosphate)	905	Itraconazole	1405	Raltegravir		
406	Clinofibrate	906	Ivabradine (hydrochloride)	1406	Raltegravir (potassium salt)		
407	Clioquinol	907	Ivacaftor	1407	Raltitrexed		
408	Clobetasol propionate	908	Ivermectin	1408	Ramatroban		
409	Clofarabine	909	Ivosidenib	1409	Ramelteon		
410	Clofazimine	910	Ixabepilone	1410	Ramipril		
411	Clofibrate	911	Ixazomib	1411	Ramosetron (Hydrochloride)		
412	Clofibric acid	912	Ixazomib citrate	1412	Ranitidine (hydrochloride)		
413	Clofoctol	913	Josamycin	1413	Ranolazine		
414	Clomiphene (citrate)	914	JQ-1	1414	Ranolazine (dihydrochloride)		
415	Clomipramine (hydrochloride)	915	Kanamycin (sulfate)	1415	Rasagiline (mesylate)		
416	Clonidine (hydrochloride)	916	Kasugamycin (hydrochloride hydrate)	1416	Rauwolscine (hydrochloride)		
417	Cloperastine fendizoate	917	Ketanserin	1417	Rebamipide		
418	Clopidogrel (hydrogen sulfate)	918	Ketanserin (tartrate)	1418	Reboxetine (mesylate)		
419	Clorprenaline hydrochloride	919	Ketoconazole	1419	Regadenoson		
420	Clotrimazole	920	Ketoprofen	1420	Regorafenib		
421	Cloxacillin (sodium monohydrate)	921	Ketorolac (tromethamine salt)	1421	Regorafenib (Hydrochloride)		
422	Clozapine	922	Ketotifen (fumarate)	1422	Regorafenib (monohydrate)		
423	Cobicistat	923	L-(-)-α-Methyldopa (hydrate)	1423	Relugolix		
424	Cobimetinib	924	L-5-Hydroxytryptophan	1424	Repaglinide		
425	Cobimetinib (hemifumarate)	925	Labetalol (hydrochloride)	1425	Reserpine		
426	Cobimetinib (racemate)	926	Lacidipine	1426	Reserpine (hydrochloride)		
427	Colchicine	927	Lactulose	1427	Resorcinol		
428	Colistin (sulfate)	928	Lafutidine	1428	Resveratrol		
429	Conivaptan (hydrochloride)	929	Lamivudine	1429	Retapamulin		
430	Cortisone	930	Lamotrigine	1430	Retinoic acid		
431	Cortisone (acetate)	931	Lanatoside C	1431	Retinol		
432	Crisaborole	932	Lansoprazole	1432	Revefenacin		
433	Crizotinib	933	Lapatinib	1433	Ribavirin		
434	Crizotinib (hydrochloride)	934	Lapatinib (ditosylate)	1434	Ribociclib		
435	Cromolyn (sodium)	935	L-Arginine	1435	Ribociclib hydrochloride		
436	Crotamiton	936	L-Arginine (hydrochloride)	1436	Ribociclib succinate		
437	Cyclandelate	937	Laropiprant	1437	Ribociclib succinate hydrate		
438	Cyclic somatostatin	938	Larotrectinib	1438	Riboflavin Tetrabutyrate		
439	Cyclobenzaprine (hydrochloride)	939	Larotrectinib sulfate	1439	Ribostamycin (sulfate)		
440	Cyclosporin A	940	L-Ascorbic acid	1440	Rifabutin		
441	Cyproheptadine (hydrochloride sesquihydrate)	941	L-Ascorbic acid sodium salt	1441	Rifampicin		
442	Cyproheptadine (hydrochloride)	942	Lasofoxifene (Tartrate)	1442	Rifapentine		
443	Cyproterone (acetate)	943	Latanoprost	1443	Rifaximin		
444	Cysteamine	944	Latrepirdine (dihydrochloride)	1444	Rilpivirine		
445	Cysteamine hydrochloride	945	L-Cysteine	1445	Riluzole		
446	Cytarabine	946	LCZ696	1446	Riluzole hydrochloride		
447	Cytidine	947	Lecithin	1447	Rimantadine (hydrochloride)		

448	Cytisinicline	948	Ledipasvir	1448	Riociguat		
449	Dabigatran etexilate	949	Ledipasvir (acetone)	1449	Ripasudil		
450	Dabigatran etexilate (mesylate)	950	Ledipasvir (D-tartrate)	1450	Risedronate (sodium)		
451	Dabrafenib	951	Leflunomide	1451	Risperidone		
452	Dabrafenib (Mesylate)	952	Lenalidomide	1452	Ritonavir		
453	Dacarbazine	953	Lenalidomide (hemihydrate)	1453	Rivaroxaban		
454	Daclatasvir	954	Lenvatinib	1454	Rivastigmine		
455	Daclatasvir (dihydrochloride)	955	L-Epinephrine (Bitartrate)	1455	Rivastigmine (tartrate)		
456	Dacomitinib	956	Lercanidipine (hydrochloride)	1456	Rocuronium (Bromide)		
457	Dalbavancin	957	Lesinurad	1457	Rofecoxib		
458	Danazol	958	Lesinurad (sodium)	1458	Roflumilast		
459	Danofloxacin (mesylate)	959	Letrozole	1459	Rolapitant		
460	Danoprevir	960	Leuprolide Acetate	1460	Ronidazole		
461	Danthron	961	Levamisole (hydrochloride)	1461	Ropinirole (hydrochloride)		
462	Dantrolene (sodium hemiheptahydrate)	962	Levetiracetam	1462	Ropivacaine (hydrochloride monohydrate)		
463	Dapagliflozin	963	Levobetaxolol (hydrochloride)	1463	Ropivacaine hydrochloride		
464	Dapagliflozin ((2S)-1,2-propanediol, hydrate)	964	Levobunolol (hydrochloride)	1464	Rosiglitazone		
465	Dapiprazole (hydrochloride)	965	Levobupivacaine (hydrochloride)	1465	Rosiglitazone (maleate)		
466	Dapoxetine (hydrochloride)	966	Levocarnitine propionate (hydrochloride)	1466	Rosuvastatin (Calcium)		
467	Dapsone	967	Levodropropizine	1467	Rotigotine		
468	Daptomycin	968	Levofloxacin	1468	Rotigotine (Hydrochloride)		
469	Darifenacin (hydrobromide)	969	Levofloxacin (hydrate)	1469	Rotundine		
470	Darunavir	970	Levoleucovorin (Calcium)	1470	Roxadustat		
471	Darunavir (Ethanolate)	971	Levosimendan	1471	Roxatidine (Acetate Hydrochloride)		
472	Dasabuvir	972	Levosulpiride	1472	Roxithromycin		
473	Dasatinib	973	L-Glutamic acid monosodium salt	1473	Rucaparib (Camsylate)		
474	Dasatinib (hydrochloride)	974	L-Glutamine	1474	Rucaparib (phosphate)		
475	Daunorubicin (Hydrochloride)	975	Lidocaine	1475	Rufinamide		
476	D-Cycloserine	976	Lidocaine (hydrochloride)	1476	Rupatadine (Fumarate)		
477	Decamethonium (Bromide)	977	Lifitegrast	1477	Rutin		
478	Decitabine	978	Limaprost	1478	Ruxolitinib		
479	Decloxizine (dihydrochloride)	979	Linagliptin	1479	Ruxolitinib (phosphate)		
480	Deferasirox	980	Lincomycin (hydrochloride hydrate)	1480	S-(+)-Ketoprofen		
481	Deferasirox (Fe3+ chelate)	981	Linezolid	1481	Sacubitril		
482	Deferiprone	982	Liothyronine	1482	Sacubitril hemicalcium salt		
483	Deferoxamine (mesylate)	983	Liothyronine (sodium)	1483	Safinamide		
484	Deflazacort	984	Lisinopril (dihydrate)	1484	Salbutamol (hemisulfate)		
485	Degarelix	985	L-Lactic acid	1485	Salicylic acid		
486	Dehydrocholic acid	986	Lodenafil	1486	Salmeterol		
487	Delafloxacin (meglumine)	987	Lodoxamide	1487	Salmeterol (xinafoate)		
488	Delamanid	988	Lodoxamide (tromethamine)	1488	Salsalate		
489	Delavirdine (mesylate)	989	Lofexidine	1489	Santonin		
490	Demecarium Bromide	990	Lomefloxacin (hydrochloride)	1490	Sapacitabine		
491	Demeclocycline (hydrochloride)	991	Lomerizine dihydrochloride	1491	Sapropterin (dihydrochloride)		
492	Deoxycholic acid	992	Lomitapide	1492	Saquinavir		
493	Deoxycholic acid sodium salt	993	Lomustine	1493	Saquinavir (Mesylate)		
494	Deoxycorticosterone (acetate)	994	Loperamide (hydrochloride)	1494	Sarpogrelate (hydrochloride)		
495	Dequalinium (Chloride)	995	Lopinavir	1495	Saxagliptin		
496	Deracoxib	996	Loratadine	1496	Scopolamine (hydrobromide)		
497	Desipramine hydrochloride	997	Lorlatinib	1497	Scopolamine butylbromide		

498	Desloratadine	998	L-Ornithine	1498	Secnidazole		
499	Desogestrel	999	Lornoxicam	1499	Selamectin		
500	Desonide	1000	Losartan	1500	Selenomethionine		
