# Supplementary material for: Phenotypical screening on metastatic PRCC-TFE3 fusion translocation renal cell carcinoma organoids reveals potential therapeutic agents
Source: Clin Transl Oncol. 2022 Feb 3;24(7):1333–46. doi: 10.1007/s12094-021-02774-8 (PMC9192364; doi:10.1007/s12094-021-02774-8)
Supplement: Supplementary file 3 — Supplementary file3 (PDF 45 KB) [file 12094_2021_2774_MOESM3_ESM.pdf]

Supplementary Data 2. The list of selected 57 non-chemotherapy drugs

No	Product Name	No	Product Name
1	Abacavir	31	Lenalidomide (hemihydrate)
2	Acebutolol (hydrochloride)	32	Loteprednol Etabonate
3	Acitretin	33	L-Thyroxine (sodium salt pentahydrate)
4	Axitnib	34	Lubiprostone
5	Balsalazide	35	Meclofenoxate (hydrochloride)
6	Belinostat	36	Metergoline
7	Bortezomib	37	Mexiletine (hydrochloride)
8	Cabozantinib	38	Midostaurin
9	Carbazochrome (sodium sulfonate)	39	Miltefosine
10	Ceritinib dihydrochloride	40	Mirabegron
11	Chlorphenoxamine	41	Nabumetone
12	Cobimetinib (racemate)	42	Niclosamide
13	Cobimetinib (racemate)	43	Otilonium (bromide)
14	crotinib	44	Ouabain (Octahydrate)
15	Dacomitinib	45	Panobinostat
16	Diphenylpyraline (hydrochloride)	46	Phenytoin
17	Ebastine	47	Ponatinib
18	Elafibranor	48	Pyrvinium pamoate
19	Emetine (dihydrochloride)	49	Rivastigmine (tartrate)
20	Entrectinib	50	Rotundine
21	Estradiol (cypionate)	51	Salmeterol (xinafoate)
22	Fimasartan	52	Setiptiline
23	Fostamatinib Disodium	53	Sunitinib
24	Homoharringtonine	54	Teneligliptin
25	Hydrocortisone 17-butyrate	55	Upadacitinib
26	Ingenol	56	Vandetanib
27	Ixazomib	57	Yohimbine (Hydrochloride)
28	Ixazomib citrate		
29	JQ-1		
30	Ketanserin		
