# Supplementary material for: Phenotypical screening on metastatic PRCC-TFE3 fusion translocation renal cell carcinoma organoids reveals potential therapeutic agents
Source: Clin Transl Oncol. 2022 Feb 3;24(7):1333–46. doi: 10.1007/s12094-021-02774-8 (PMC9192364; doi:10.1007/s12094-021-02774-8)
Supplement: Supplementary file 4 — Supplementary file4 (PDF 109 KB) [file 12094_2021_2774_MOESM4_ESM.pdf]

Supplementary Data 3. Differential gene lists of the organoid after treatments according to the RNA-seq

Upgraded Genes of Axitinib-High Dose Group (10)					
No	Gene ID	Gene Symbol	No	Gene ID	Gene Symbol
1	1028	'CDKN1C'	6	2354	'FOSB'
2	1277	'COL1A1'	7	407977	'TNFSF12-TNFSF13'
3	1356	'CP'	8	4586	'MUC5AC'
4	2252	'FGF7'	9	5764	'PTN'
5	2335	'FN1'	10	92815	'H2AW'

Downpgraded Genes of Axitinib-High Dose Group (1)		
No	Gene ID	Gene Symbol
1	100529241	HSPE1-MOB4'

Upgraded Genes of JQ1-High Dose Group (8)					
No	Gene ID	Gene Symbol	No	Gene ID	Gene Symbol
1	1490	'CCN2'	5	4318	'MMP9'
2	2121	'EVC'	6	60676	'PAPPA2'
3	23089	'PEG10'	7	79057	'PRRG3'
4	3553	'IL1B'	8	8363	'H4C11'

Downpgraded Genes of JQ1-High Dose Group (28)											
No	Gene ID	Gene Symbol	No	Gene ID	Gene Symbol	No	Gene ID	Gene Symbol	No	Gene ID	Gene Symbol
1	10158	'PDZK1IP1'	8	3934	'LCN2'	15	6398	'SECTM1'	22	80115	'BAIAP2L2'
2	10786	'SLC17A3'	9	4057	'LTF'	16	6568	'SLC17A1'	23	80149	'ZC3H12A'
3	12	'SERPINA3'	10	407977	'TNFSF12-TNFSF13'	17	6750	'SST'	24	81557	'MAGED4B'
4	2243	'FGA'	11	5265	'SERPINA1'	18	7127	'TNFAIP2'	25	84465	'MEGF11'
5	2919	'CXCL1'	12	6288	'SAA1'	19	718	'C3'	26	84929	'FIBCD1'
6	2920	'CXCL2'	13	6289	'SAA2'	20	720	'C4A'	27	9075	'CLDN2'
7	3868	'KRT16'	14	6347	'CCL2'	21	728441	'GGT2'	28	9476	'NAPSA'

Upgraded Genes of Crizotinib-High Dose Group (364)					
No	Gene ID	Gene Symbol	No	Gene ID	Gene Symbol
1	10000	'AKT3'	201	440345	'NPIPB4'
2	100037417	'DDTL'	202	440503	'PLIN5'
3	100132247	'NPIPB5'	203	4548	'MTR'
4	100137049	'PLA2G4B'	204	4616	'GADD45B'
5	10018	'BCL2L11'	205	4649	'MYO9A'
6	100288695	'LIMS4'	206	472	'ATM'
7	100293534	'C4B_2'	207	4739	'NEDD9'
8	100462977	'MTRNR2L1'	208	4779	'NFE2L1'
9	100462981	'MTRNR2L2'	209	4881	'NPR1'
10	100462983	'MTRNR2L3'	210	5047	'PAEP'
11	100463285	'MTRNR2L4'	211	50486	'G0S2'
12	100463482	'MTRNR2L6'	212	5074	'PAWR'
13	100463486	'MTRNR2L8'	213	51005	'AMDHD2'
14	100463487	'MTRNR2L9'	214	51176	'LEF1'
15	100463488	'MTRNR2L10'	215	51309	'ARMCX1'
16	100526783	'ARPIN-AP3S2'	216	51365	'PLA1A'
17	100529241	'HSPE1-MOB4'	217	51454	'GULP1'
18	101060226	'NBPF19'	218	5166	'PDK4'
19	10157	'AASS'	219	51696	'HECA'
20	10158	'PDZK1IP1'	220	5208	'PFKFB2'
21	10170	'DHRS9'	221	523	'ATP6V1A'
22	102723996	'LOC102723996'	222	5292	'PIM1'

Downgraded Genes of Crizotinib-High Dose Group (671)											
No	Gene ID	Gene Symbol	No	Gene ID	Gene Symbol	No	Gene ID	Gene Symbol	No	Gene ID	Gene Symbol
1	1001	'CDH3'	201	2678	'GGT1'	401	55355	'HJURP'	601	84634	'KISS1R'
2	10024	'TROAP'	202	2683	'B4GALT1'	402	55450	'CAMK2N1'	602	84675	'TRIM55'
3	10050	'SLC17A4'	203	2702	'GJA5'	403	5549	'PRELP'	603	84790	'TUBA1C'
4	100505385	'IQCJ-SCHIP1'	204	2703	'GJA8'	404	55723	'ASF1B'	604	84915	'FAM222A'
5	100527963	'PMF1-BGLAP'	205	2707	'GJB3'	405	5578	'PRKCA'	605	84929	'FIBCD1'
6	100528064	'NEDD8-MDP1'	206	27143	'PALD1'	406	55790	'CSGALNACT1'	606	84952	'CGNL1'
7	10058	'ABCB6'	207	27286	'SRPX2'	407	55806	'HR'	607	8497	'PPFIA4'
8	1009	'CDH11'	208	2731	'GLDC'	408	55872	'PBK'	608	8503	'PIK3R3'
9	10103	'TSPAN1'	209	27329	'ANGPTL3'	409	55879	'GABRQ'	609	8522	'GAS7'
10	10112	'KIF20A'	210	27345	'KCNMB4'	410	5596	'MAPK4'	610	8543	'LMO4'
11	10203	'CALCRL'	211	2743	'GLRB'	411	55966	'AJAP1'	611	8573	'CASK'
12	10220	'GDF11'	212	2810	'SFN'	412	5606	'MAP2K3'	612	8635	'RNASET2'
13	102723360	'LOC102723360'	213	283455	'KSR2'	413	5608	'MAP2K6'	613	866	'SERPINA6'
14	102724594	'U2AF1L5'	214	284	'ANGPT1'	414	56245	'C21orf62'	614	8701	'DNAH11'
15	1028	'CDKN1C'	215	284040	'CDRT4'	415	56265	'CPXM1'	615	871	'SERPINH1'
16	10290	'SPEG'	216	284297	'SSC5D'	416	5653	'KLK6'	616	8745	'ADAM23'
17	10376	'TUBA1B'	217	284403	'WDR62'	417	5654	'HTRA1'	617	8771	'TNFRSF6B'
18	10382	'TUBB4A'	218	284716	'RIMKLA'	418	56603	'CYP26B1'	618	8796	'SCEL'
19	10383	'TUBB4B'	219	285016	'ALKAL2'	419	56649	'TMPRSS4'	619	8817	'FGF18'
20	10398	'MYL9'	220	285489	'DOK7'	420	56944	'OLFML3'	620	8828	'NRP2'
21	10406	'WFDC2'	221	2893	'GRIA4'	421	56992	'KIF15'	621	890	'CCNA2'
22	10409	'BASP1'	222	28969	'BZW2'	422	57106	'NAT14'	622	8900	'CCNA1'

23	1030	'CDKN2B'	223	5364	'PLXNB1'
24	10350	'ABCA9'	224	54206	'ERRFI1'
25	10370	'CITED2'	225	54463	'RETREG1'
26	10417	'SPON2'	226	54507	'ADAMTSL4'
27	10457	'GPNMB'	227	54520	'CCDC93'
28	10493	'VAT1'	228	54557	'SGTB'
29	105379547	'LOC105379547'	229	54600	'UGT1A9'
30	10579	'TACC2'	230	54741	'LEPROT'
31	10628	'TXNIP'	231	54762	'GRAMD1C'
32	10826	'FAXDC2'	232	54800	'KLHL24'
33	10840	'ALDH1L1'	233	54893	'MTMR10'
34	10891	'PPARGC1A'	234	54913	'RPP25'
35	10924	'SMPDL3A'	235	55102	'ATG2B'
36	10966	'RAB40B'	236	55238	'SLC38A7'
37	11056	'DDX52'	237	55251	'PCMTD2'
38	11067	'DEPP1'	238	55281	'TMEM140'
39	11076	'TPPP'	239	55289	'ACOXL'
40	1112	'FOXN3'	240	55326	'AGPAT5'
41	1116	'CHI3L1'	241	55356	'SLC22A15'
42	1122	'CHML'	242	55616	'ASAP3'
43	112268437	'LOC112268437'	243	55638	'SYBU'
44	112849	'L3HYPDH'	244	5565	'PRKAB2'
45	113455421	'DERPC'	245	56548	'CHST7'
46	113791	'PIK3IP1'	246	56670	'SUCNR1'
47	114876	'OSBPL1A'	247	56957	'OTUD7B'
48	1154	'CISH'	248	56997	'COQ8A'
49	116150	'NUS1'	249	57035	'RSRP1'
50	117248	'GALNT15'	250	5733	'PTGER3'
51	118881	'COMTD1'	251	57475	'PLEKHH1'
52	1201	'CLN3'	252	57486	'NLN'
53	1241	'LTB4R'	253	57494	'RIMKLB'
54	124961	'ZFP3'	254	57509	'MTUS1'
55	1268	'CNR1'	255	57600	'FNIP2'
56	127018	'LYPLAL1'	256	57687	'VAT1L'
57	130271	'PLEKHH2'	257	57728	'WDR19'
58	1316	'KLF6'	258	5797	'PTPRM'
59	134285	'TMEM171'	259	58528	'RRAGD'
60	135398	'C6orf141'	260	5934	'RBL2'
61	137075	'CLDN23'	261	5950	'RBP4'
62	1374	'CPT1A'	262	5973	'RENBP'
63	137872	'ADHFE1'	263	60468	'BACH2'
64	139221	'PWWP3B'	264	606495	'CYB5RL'
65	140453	'MUC17'	265	6097	'RORC'
66	145741	'C2CD4A'	266	6324	'SCN1B'
67	1462	'VCAN'	267	6398	'SECTM1'
68	1469	'CST1'	268	64123	'ADGRL4'
69	150465	'TTL'	269	643338	'C15orf62'
70	151230	'KLHL23'	270	64342	'HS1BP3'
71	151449	'GDF7'	271	643707	'GOLGA6L4'
72	151525	'WDSUB1'	272	6484	'ST3GAL4'

23	10418	'SPON1'	223	2904	'GRIN2B'	423	57224	'NHSL1'	623	891	'CCNB1'
24	10460	'TACC3'	224	29108	'PYCARD'	424	5724	'PTAFR'	624	8913	'CACNA1G'
25	1050	'CEBPA'	225	29128	'UHRF1'	425	5728	'PTEN'	625	89765	'RSPH1'
26	10509	'SEMA4B'	226	2938	'GSTA1'	426	57333	'RCN3'	626	8991	'SELENBP1'
27	10535	'RNASEH2A'	227	2940	'GSTA3'	427	5737	'PTGFR'	627	89958	'SAPCD2'
28	10536	'P3H3'	228	2944	'GSTM1'	428	57405	'SPC25'	628	90293	'KLHL13'
29	10551	'AGR2'	229	2949	'GSTM5'	429	5742	'PTGS1'	629	9052	'GPRC5A'
30	10561	'IFI44'	230	2954	'GSTZ1'	430	5743	'PTGS2'	630	9055	'PRC1'
31	10615	'SPAG5'	231	29923	'HILPDA'	431	5745	'PTH1R'	631	9087	'TMSB4Y'
32	1063	'CENPF'	232	29943	'PADI1'	432	57484	'RNF150'	632	9088	'PKMYT1'
33	10631	'POSTN'	233	29968	'PSAT1'	433	57565	'KLHL14'	633	91179	'SCARF2'
34	10637	'LEFTY1'	234	30001	'ERO1A'	434	576	'ADGRB2'	634	9133	'CCNB2'
35	10786	'SLC17A3'	235	30008	'EFEMP2'	435	5764	'PTN'	635	91461	'PKDCC'
36	10858	'CYP46A1'	236	30011	'SH3KBP1'	436	58	'ACTA1'	636	91522	'COL23A1'
37	10875	'FGL2'	237	3081	'HGD'	437	5802	'PTPRS'	637	91683	'SYT12'
38	109504726	'ERV3-1-ZNF117'	238	3099	'HK2'	438	5806	'PTX3'	638	9170	'LPAR2'
39	10964	'IFI44L'	239	3161	'HMMR'	439	5831	'PYCR1'	639	9173	'IL1RL1'
40	10974	'ADIRF'	240	3164	'NR4A1'	440	58504	'ARHGAP22'	640	9201	'DCLK1'
41	11004	'KIF2C'	241	3212	'HOXB2'	441	59269	'HIVEP3'	641	9212	'AURKB'
42	11007	'CCDC85B'	242	3215	'HOXB5'	442	59350	'RXFP1'	642	92291	'CAPN13'
43	11065	'UBE2C'	243	3218	'HOXB8'	443	595	'CCND1'	643	9232	'PTTG1'
44	111	'ADCY5'	244	3234	'HOXD8'	444	5997	'RGS2'	644	9244	'CRLF1'
45	11117	'EMILIN1'	245	3280	'HES1'	445	60598	'KCNK15'	645	9249	'DHRS3'
46	11135	'CDC42EP1'	246	3305	'HSPA1L'	446	60676	'PAPPA2'	646	92815	'H2AW'
47	11156	'PTP4A3'	247	332	'BIRC5'	447	60681	'FKBP10'	647	9388	'LIPG'
48	11167	'FSTL1'	248	333	'APLP1'	448	6142	'RPL18A'	648	94	'ACVRL1'
49	11170	'FAM107A'	249	333926	'PPM1J'	449	6206	'RPS12'	649	94033	'FTMT'
50	112267859	'LOC112267859'	250	337876	'CHSY3'	450	623	'BDKRB1'	650	94120	'SYTL3'
51	112399	'EGLN3'	251	339479	'BRINP3'	451	6241	'RRM2'	651	9415	'FADS2'
52	11240	'PADI2'	252	3399	'ID3'	452	6273	'S100A2'	652	9427	'ECEL1'
53	11259	'FILIP1L'	253	340547	'VSIG1'	453	6275	'S100A4'	653	9435	'CHST2'
54	112694756	'LOC112694756'	254	3426	'CFI'	454	6280	'S100A9'	654	948	'CD36'
55	113130	'CDCA5'	255	342667	'STAC2'	455	631	'BFSP1'	655	9493	'KIF23'
56	114788	'CSMD3'	256	343069	'HNRNPCL1'	456	633	'BGN'	656	9510	'ADAMTS1'
57	114794	'ELFN2'	257	347688	'TUBB8'	457	6332	'SCN7A'	657	9514	'GAL3ST1'
58	115207	'KCTD12'	258	3485	'IGFBP2'	458	6344	'SCTR'	658	954	'ENTPD2'
59	115265	'DDIT4L'	259	3486	'IGFBP3'	459	6347	'CCL2'	659	9590	'AKAP12'
60	115290	'FBXO17'	260	3488	'IGFBP5'	460	6382	'SDC1'	660	9607	'CARTPT'
61	115677	'NOSTRIN'	261	3489	'IGFBP6'	461	63917	'GALNT11'	661	9636	'ISG15'
62	115703	'ARHGAP33'	262	349667	'RTN4RL2'	462	64073	'C19orf33'	662	9700	'ESPL1'
63	116039	'OSR2'	263	353322	'ANKRD37'	463	64077	'LHPP'	663	9746	'CLSTN3'
64	116154	'PHACTR3'	264	3547	'IGSF1'	464	64101	'LRRC4'	664	9768	'PCLAF'
65	116372	'LYPD1'	265	3569	'IL6'	465	641455	'POTEM'	665	9787	'DLGAP5'
66	116844	'LRG1'	266	3671	'ISLR'	466	64151	'NCAPG'	666	983	'CDK1'
67	117581	'TWIST2'	267	3678	'ITGA5'	467	6422	'SFRP1'	667	990	'CDC6'
68	118663	'BTBD16'	268	3694	'ITGB6'	468	64236	'PDLIM2'	668	9902	'MRC2'
69	1240	'CMKLR1'	269	3708	'ITPR1'	469	6446	'SGK1'	669	991	'CDC20'
70	124930	'ANKRD13B'	270	3730	'ANOS1'	470	6447	'SCG5'	670	9982	'FGFBP1'
71	125113	'KRT222'	271	3732	'CD82'	471	6469	'SHH'	671	9985	'REC8'
72	126129	'CPT1C'	272	374393	'FAM111B'	472	646960	'PRSS56'			

73	151742	'PPM1L'	273	6505	'SLC1A1'
74	153222	'CREBRF'	274	651746	'ANKRD33B'
75	1577	'CYP3A5'	275	6519	'SLC3A1'
76	158471	'PRUNE2'	276	65250	'CPLANE1'
77	1593	'CYP27A1'	277	6526	'SLC5A3'
78	1604	'CD55'	278	654463	'FER1L6'
79	160851	'DGKH'	279	66008	'TRAK2'
80	162417	'NAGS'	280	6648	'SOD2'
81	1634	'DCN'	281	6755	'SSTR5'
82	1646	'AKR1C2'	282	687	'KLF9'
83	166824	'RASSF6'	283	6990	'DYNLT3'
84	169792	'GLIS3'	284	7035	'TFPI'
85	1831	'TSC22D3'	285	7049	'TGFBR3'
86	1852	'DUSP9'	286	7056	'THBD'
87	192670	'AGO4'	287	7099	'TLR4'
88	1956	'EGFR'	288	710	'SERPING1'
89	196446	'MYRFL'	289	7145	'TNS1'
90	201163	'FLCN'	290	716	'C1S'
91	202333	'CMYA5'	291	7188	'TRAF5'
92	203523	'ZNF449'	292	7248	'TSC1'
93	2065	'ERBB3'	293	728047	'GOLGA8O'
94	2081	'ERN1'	294	728577	'CNTNAP3B'
95	2170	'FABP3'	295	728741	'NPIPB6'
96	2180	'ACSL1'	296	7296	'TXNRD1'
97	219736	'STOX1'	297	734	'OSGIN2'
98	2202	'EFEMP1'	298	7552	'ZNF711'
99	220213	'OTUD1'	299	7704	'ZBTB16'
100	220441	'RNF152'	300	7763	'ZFAND5'
101	221	'ALDH3B1'	301	7780	'SLC30A2'
102	221692	'PHACTR1'	302	7852	'CXCR4'
103	221785	'ZSCAN25'	303	79037	'PVRIG'
104	221981	'THSD7A'	304	79616	'CCNJL'
105	224	'ALDH3A2'	305	79794	'SPRING1'
106	2263	'FGFR2'	306	79817	'MOB3B'
107	2267	'FGL1'	307	79901	'CYBRD1'
108	2289	'FKBP5'	308	79937	'CNTNAP3'
109	22909	'FAN1'	309	80005	'DOCK5'
110	22941	'SHANK2'	310	80031	'SEMA6D'
111	23052	'ENDOD1'	311	80162	'PGGHG'
112	23117	'NPIPB3'	312	80176	'SPSB1'
113	23133	'PHF8'	313	80262	'C16orf70'
114	23138	'N4BP3'	314	80325	'ABTB1'
115	23175	'LPIN1'	315	80763	'SPX'
116	23277	'CLUH'	316	81035	'COLEC12'
117	23308	'ICOSLG'	317	81606	'LBH'
118	23338	'JADE2'	318	81848	'SPRY4'
119	23345	'SYNE1'	319	83937	'RASSF4'
120	23371	'TNS2'	320	83992	'CTTNBP2'
121	23405	'DICER1'	321	84085	'FBXO30'
122	23411	'SIRT1'	322	8411	'EEA1'

73	1271	'CNTFR'	273	374569	'ASPG'	473	64699	'TMPRSS3'			
74	1277	'COL1A1'	274	3758	'KCNJ1'	474	64856	'VWA1'			
75	1278	'COL1A2'	275	3772	'KCNJ15'	475	64922	'LRRC19'			
76	1281	'COL3A1'	276	377677	'CA13'	476	64943	'NT5DC2'			
77	128178	'EDARADD'	277	3778	'KCNMA1'	477	6495	'SIX1'			
78	128239	'IQGAP3'	278	3784	'KCNQ1'	478	6508	'SLC4A3'			
79	1289	'COL5A1'	279	3833	'KIFC1'	479	65108	'MARCKSL1'			
80	1290	'COL5A2'	280	3855	'KRT7'	480	6513	'SLC2A1'			
81	1292	'COL6A2'	281	3861	'KRT14'	481	6525	'SMTN'			
82	1295	'COL8A1'	282	3868	'KRT16'	482	653269	'POTEI'			
83	1298	'COL9A2'	283	387082	'SUMO4'	483	653319	'KIAA0895L'			
84	130	'ADH6'	284	3872	'KRT17'	484	653598	'PPIAL4C'			
85	130340	'AP1S3'	285	387509	'GPR153'	485	653659	'TMEM183B'			
86	130497	'OSR1'	286	387695	'C10orf99'	486	654364	'NME1-NME2'			
87	1307	'COL16A1'	287	3880	'KRT19'	487	6546	'SLC8A1'			
88	131	'ADH7'	288	388135	'INSYN1'	488	6557	'SLC12A1'			
89	133688	'UGT3A1'	289	388610	'TRNP1'	489	6565	'SLC15A2'			
90	1356	'CP'	290	389332	'SMIM32'	490	6568	'SLC17A1'			
91	1373	'CPS1'	291	389376	'SFTA2'	491	6608	'SMO'			
92	138065	'RNF183'	292	389792	'IER5L'	492	6614	'SIGLEC1'			
93	140465	'MYL6B'	293	390205	'LRRC10B'	493	6623	'SNCG'			
94	140801	'RPL10L'	294	3910	'LAMA4'	494	6636	'SNRPF'			
95	140823	'ROMO1'	295	3915	'LAMC1'	495	6640	'SNTA1'			
96	1428	'CRYM'	296	3918	'LAMC2'	496	6649	'SOD3'			
97	143244	'EIF5AL1'	297	392490	'FLJ44635'	497	6662	'SOX9'			
98	143903	'LAYN'	298	3956	'LGALS1'	498	6676	'SPAG4'			
99	144501	'KRT80'	299	399473	'SPRED3'	499	6678	'SPARC'			
100	144983	'HNRNPA1L2'	300	4001	'LMNB1'	500	6695	'SPOCK1'			
101	1466	'CSRP2'	301	401115	'C4orf48'	501	6696	'SPP1'			
102	146909	'KIF18B'	302	401250	'MCCD1'	502	6751	'SSTR1'			
103	147111	'NOTUM'	303	4016	'LOXL1'	503	684	'BST2'			
104	147183	'KRT25'	304	4017	'LOXL2'	504	6909	'TBX2'			
105	148398	'SAMD11'	305	402778	'IFITM10'	505	6925	'TCF4'			
106	149461	'CLDN19'	306	4037	'LRP3'	506	6941	'TCF19'			
107	150372	'NFAM1'	307	4045	'LSAMP'	507	699	'BUB1'			
108	150468	'CKAP2L'	308	4057	'LTF'	508	70	'ACTC1'			
109	150696	'PROM2'	309	407977	'TNFSF12-TNFSF13'	509	701	'BUB1B'			
110	151176	'ERFE'	310	4094	'MAF'	510	7025	'NR2F1'			
111	151354	'LRATD1'	311	4118	'MAL'	511	7031	'TFF1'			
112	151887	'CCDC80'	312	4133	'MAP2'	512	7032	'TFF2'			
113	152002	'XXYLT1'	313	4147	'MATN2'	513	7043	'TGFB3'			
114	152110	'NEK10'	314	4148	'MATN3'	514	7052	'TGM2'			
115	155006	'TMEM213'	315	4211	'MEIS1'	515	7078	'TIMP3'			
116	1572	'CYP2F1'	316	4212	'MEIS2'	516	7083	'TK1'			
117	157869	'SBSPON'	317	4232	'MEST'	517	7089	'TLE2'			
118	162466	'PHOSPHO1'	318	4282	'MIF'	518	7102	'TSPAN7'			
119	163351	'GBP6'	319	4288	'MKI67'	519	7108	'TM7SF2'			
120	164091	'PAQR7'	320	4313	'MMP2'	520	7114	'TMSB4X'			
121	165	'AEBP1'	321	440093	'H3-5'	521	7153	'TOP2A'			
122	165257	'C1QL2'	322	441054	'C4orf47'	522	7167	'TPI1'			

123	23446	'SLC44A1'	323	84166	'NLRC5'
124	23460	'ABCA6'	324	8417	'STX7'
125	23461	'ABCA5'	325	84220	'RGPD5'
126	23492	'CBX7'	326	84260	'TCHP'
127	23516	'SLC39A14'	327	84293	'PRXL2A'
128	23530	'NNT'	328	84336	'TMEM101'
129	23657	'SLC7A11'	329	84532	'ACSS1'
130	253260	'RICTOR'	330	84750	'FUT10'
131	254427	'PROSER2'	331	84851	'TRIM52'
132	254887	'ZDHHC23'	332	84913	'ATOH8'
133	2549	'GAB1'	333	8501	'SLC43A1'
134	256764	'WDR72'	334	85027	'SMIM3'
135	25825	'BACE2'	335	8515	'ITGA10'
136	25992	'SNED1'	336	85379	'KIAA1671'
137	26	'AOC1'	337	85457	'CIPC'
138	26253	'CLEC4E'	338	8578	'SCARF1'
139	2634	'GBP2'	339	8609	'KLF7'
140	26471	'NUPR1'	340	8660	'IRS2'
141	26873	'OPLAH'	341	8722	'CTSF'
142	27106	'ARRDC2'	342	8743	'TNFSF10'
143	27303	'RBMS3'	343	89932	'PAPLN'
144	2752	'GLUL'	344	9001	'HAP1'
145	283991	'UBALD2'	345	90427	'BMF'
146	284391	'ZNF844'	346	90874	'ZNF697'
147	285268	'ZNF621'	347	9108	'MTMR7'
148	2878	'GPX3'	348	91156	'IGFN1'
149	2887	'GRB10'	349	92017	'SNX29'
150	28960	'DCPS'	350	92211	'CDHR1'
151	28968	'SLC6A16'	351	9229	'DLGAP1'
152	28970	'C11orf54'	352	924	'CD7'
153	28996	'HIPK2'	353	9476	'NAPSA'
154	28999	'KLF15'	354	9481	'SLC25A27'
155	29121	'CLEC2D'	355	9547	'CXCL14'
156	29767	'TMOD2'	356	9619	'ABCG1'
157	29799	'YPEL1'	357	96626	'LIMS3'
158	29945	'ANAPC4'	358	9674	'KIAA0040'
159	3162	'HMOX1'	359	9679	'FAM53B'
160	3176	'HNMT'	360	9762	'LZTS3'
161	32	'ACACB'	361	9771	'RAPGEF5'
162	3240	'HP'	362	9829	'DNAJC6'
163	3291	'HSD11B2'	363	9846	'GAB2'
164	339122	'RAB43'	364	9903	'KLHL21'
165	340371	'NRBP2'			
166	3437	'IFIT3'			
167	3481	'IGF2'			
168	3484	'IGFBP1'			
169	3508	'IGHMBP2'			
170	352954	'CASTOR3'			
171	3553	'IL1B'			
172	3554	'IL1R1'			

123	166614	'DCLK2'	323	4490	'MT1B'	523	7169	'TPM2'			
124	167410	'LIX1'	324	4499	'MT1M'	524	72	'ACTG2'			
125	167681	'PRSS35'	325	4599	'MX1'	525	7216	'TRO'			
126	168002	'DACT2'	326	4605	'MYBL2'	526	7262	'PHLDA2'			
127	171177	'RHOV'	327	4648	'MYO7B'	527	7272	'TTK'			
128	1742	'DLG4'	328	4684	'NCAM1'	528	7277	'TUBA4A'			
129	1757	'SARDH'	329	4784	'NFIX'	529	727897	'MUC5B'			
130	1839	'HBEGF'	330	4830	'NME1'	530	728378	'POTEF'			
131	1848	'DUSP6'	331	4833	'NME4'	531	7291	'TWIST1'			
132	1869	'E2F1'	332	4856	'CCN3'	532	7298	'TYMS'			
133	1879	'EBF1'	333	4860	'PNP'	533	7314	'UBB'			
134	1947	'EFNB1'	334	4880	'NPPC'	534	7348	'UPK1B'			
135	202151	'RANBP3L'	335	4900	'NRGN'	535	7351	'UCP2'			
136	2022	'ENG'	336	493869	'GPX8'	536	7412	'VCAM1'			
137	2023	'ENO1'	337	4969	'OGN'	537	7422	'VEGFA'			
138	2041	'EPHA1'	338	5025	'P2RX4'	538	7425	'VGF'			
139	2043	'EPHA4'	339	5031	'P2RY6'	539	7431	'VIM'			
140	2047	'EPHB1'	340	5042	'PABPC3'	540	7436	'VLDLR'			
141	2049	'EPHB3'	341	50617	'ATP6V0A4'	541	7476	'WNT7A'			
142	2066	'ERBB4'	342	50805	'IRX4'	542	7477	'WNT7B'			
143	2146	'EZH2'	343	50861	'STMN3'	543	7498	'XDH'			
144	2182	'ACSL4'	344	5091	'PC'	544	762	'CA4'			
145	2191	'FAP'	345	5104	'SERPINA5'	545	768	'CA9'			
146	2199	'FBLN2'	346	5105	'PCK1'	546	771	'CA12'			
147	221527	'ZBTB12'	347	51087	'YBX2'	547	7846	'TUBA1A'			
148	2219	'FCN1'	348	51129	'ANGPTL4'	548	7850	'IL1R2'			
149	222962	'SLC29A4'	349	51162	'EGFL7'	549	7851	'MALL'			
150	2256	'FGF11'	350	5118	'PCOLCE'	550	7857	'SCG2'			
151	226	'ALDOA'	351	51200	'CPA4'	551	7867	'MAPKAPK3'			
152	2261	'FGFR3'	352	51226	'COPZ2'	552	79019	'CENPM'			
153	2264	'FGFR4'	353	5125	'PCSK5'	553	79154	'DHRS11'			
154	2274	'FHL2'	354	5129	'CDK18'	554	79366	'HMGN5'			
155	229	'ALDOB'	355	51303	'FKBP11'	555	79411	'GLB1L'			
156	2290	'FOXG1'	356	51310	'SLC22A17'	556	79413	'ZBED2'			
157	22943	'DKK1'	357	51330	'TNFRSF12A'	557	79589	'RNF128'			
158	22974	'TPX2'	358	51364	'ZMYND10'	558	79679	'VTCN1'			
159	22998	'LIMCH1'	359	51450	'PRRX2'	559	79729	'SH3D21'			
160	230	'ALDOC'	360	51473	'DCDC2'	560	79733	'E2F8'			
161	23024	'PDZRN3'	361	51512	'GTSE1'	561	79801	'SHCBP1'			
162	2304	'FOXE1'	362	51514	'DTL'	562	79850	'TLCD3A'			
163	2305	'FOXM1'	363	5159	'PDGFRB'	563	79899	'PRR5L'			
164	23129	'PLXND1'	364	51614	'ERGIC3'	564	79935	'CCNP'			
165	2318	'FLNC'	365	5163	'PDK1'	565	80115	'BAIAP2L2'			
166	23397	'NCAPH'	366	51661	'FKBP7'	566	80144	'FRAS1'			
167	23414	'ZFPM2'	367	5168	'ENPP2'	567	80164	'PRR36'			
168	2348	'FOLR1'	368	5176	'SERPINF1'	568	80326	'WNT10A'			
169	2353	'FOS'	369	5209	'PFKFB3'	569	80349	'WDR61'			
170	2354	'FOSB'	370	5210	'PFKFB4'	570	80727	'TTYH3'			
171	23600	'AMACR'	371	5223	'PGAM1'	571	80758	'PRR7'			
172	23616	'SH3BP1'	372	5230	'PGK1'	572	81557	'MAGED4B'			

173	3570	'IL6R'			
174	3572	'IL6ST'			
175	360	'AQP3'			
176	3643	'INSR'			
177	3656	'IRAK2'			
178	3667	'IRS1'			
179	3707	'ITPKB'			
180	3748	'KCNC3'			
181	375057	'STUM'			
182	375775	'PNPLA7'			
183	384	'ARG2'			
184	387680	'WASHC2A'			
185	387700	'SLC16A12'			
186	387758	'FIBIN'			
187	387914	'SHISA2'			
188	388403	'YPEL2'			
189	389072	'PLEKHM3'			
190	389432	'SAMD5'			
191	389434	'IYD'			
192	390077	'OR52N2'			
193	3987	'LIMS1'			
194	4121	'MAN1A1'			
195	4131	'MAP1B'			
196	415	'ARSL'			
197	4217	'MAP3K5'			
198	4254	'KITLG'			
199	4301	'AFDN'			
200	4318	'MMP9'			

173	23705	'CADM1'	373	5265	'SERPINA1'	573	81618	'ITM2C'			
174	24	'ABCA4'	374	5284	'PIGR'	574	81620	'CDT1'			
175	24137	'KIF4A'	375	5321	'PLA2G4A'	575	827	'CAPN6'			
176	24147	'FJX1'	376	5332	'PLCB4'	576	8291	'DYSF'			
177	245812	'CNPY4'	377	5347	'PLK1'	577	8309	'ACOX2'			
178	249	'ALPL'	378	5351	'PLOD1'	578	8318	'CDC45'			
179	2533	'FYB1'	379	5360	'PLTP'	579	8324	'FZD7'			
180	2535	'FZD2'	380	5376	'PMP22'	580	8329	'H2AC13'			
181	2537	'IFI6'	381	53827	'FXYD5'	581	8341	'H2BC15'			
182	254827	'NAALADL2'	382	5396	'PRRX1'	582	83439	'TCF7L1'			
183	254863	'TMEM256'	383	54039	'PCBP3'	583	8344	'H2BC6'			
184	2556	'GABRA3'	384	5413	'SEPTIN5'	584	8355	'H3C8'			
185	256714	'MAP7D2'	385	54437	'SEMA5B'	585	83648	'FAM167A'			
186	25837	'RAB26'	386	54443	'ANLN'	586	83715	'ESPN'			
187	2584	'GALK1'	387	54541	'DDIT4'	587	8382	'NME5'			
188	25890	'ABI3BP'	388	54843	'SYTL2'	588	83872	'HMCN1'			
189	25911	'DPCD'	389	54848	'ARHGEF38'	589	83982	'IFI27L2'			
190	25928	'SOSTDC1'	390	54857	'GDPD2'	590	84058	'WDR54'			
191	259307	'IL4I1'	391	54976	'C20orf27'	591	84152	'PPP1R1B'			
192	2597	'GAPDH'	392	54997	'TESC'	592	8424	'BBOX1'			
193	26002	'MOXD1'	393	55061	'SUSD4'	593	84296	'GINS4'			
194	26085	'KLK13'	394	5507	'PPP1R3C'	594	84300	'UQCC2'			
195	26207	'PITPNC1'	395	55165	'CEP55'	595	8436	'CAVIN2'			
196	26227	'PHGDH'	396	55195	'CCDC198'	596	8437	'RASAL1'			
197	26292	'MYCBP'	397	5524	'PTPA'	597	84465	'MEGF11'			
198	26353	'HSPB8'	398	55304	'SPTLC3'	598	8447	'DOC2B'			
199	26521	'TIMM8B'	399	55329	'MNS1'	599	84557	'MAP1LC3A'			
200	26579	'MYEOV'	400	55351	'STK32B'	600	84570	'COL25A1'			
