# Supplementary material for: Phenotypical screening on metastatic PRCC-TFE3 fusion translocation renal cell carcinoma organoids reveals potential therapeutic agents
Source: Clin Transl Oncol. 2022 Feb 3;24(7):1333–46. doi: 10.1007/s12094-021-02774-8 (PMC9192364; doi:10.1007/s12094-021-02774-8)
Supplement: Supplementary file 5 — Supplementary file5 (PDF 95 KB) [file 12094_2021_2774_MOESM5_ESM.pdf]

Supplementary Data 4. The list of 232 autophagy-related genes

No	Gene ID	Gene Name	Symbol	No	Gene ID	Gene Name	Symbol	No	Gene ID	Gene Name	Symbol
1	55626	autophagy/beclin-1 regulator 1	AMBRA1	81	1981	eukaryotic translation initiation factor 4 gamma, 1	EIF4G1	161	3084	neuregulin 1	NRG1
2	8542	apolipoprotein L, 1	APOL1	82	2064	v-erb-b2 erythroblastic leukemia viral oncogene homolog 2, neuro/glioblastoma derived oncogene homolog (avian)	ERBB2	162	9542	neuregulin 2	NRG2
3	405	aryl hydrocarbon receptor nuclear translocator	ARNT	83	2081	endoplasmic reticulum to nucleus signaling 1	ERN1	163	10718	neuregulin 3	NRG3
4	410	arylsulfatase A	ARSA	84	30001	ERO1-like (S. cerevisiae)	ERO1L	164	5034	prolyl 4-hydroxylase, beta polypeptide	P4HB
5	411	arylsulfatase B	ARSB	85	8772	Fas (TNFRSF6)-associated via death domain	FADD	165	5071	Parkinson disease (autosomal recessive, juvenile) 2, parkin	PARK2
6	468	activating transcription factor 4 (tax-responsive enhancer element B67)	ATF4	86	55578	family with sequence similarity 48, member A	FAM48A	166	142	poly (ADP-ribose) polymerase 1	PARP1
7	22926	activating transcription factor 6	ATF6	87	355	Fas (TNF receptor superfamily, member 6)	FAS	167	8682	phosphoprotein enriched in astrocytes 15	PEA15
8	83734	ATG10 autophagy related 10 homolog (S. cerevisiae)	ATG10	88	2280	FK506 binding protein 1A, 12kDa	FKBP1A	168	27043	proline, glutamate and leucine rich protein 1	PELP1
9	9140	ATG12 autophagy related 12 homolog (S. cerevisiae)	ATG12	89	2281	FK506 binding protein 1B, 12.6 kDa	FKBP1B	169	5195	peroxisomal biogenesis factor 14	PEX14
10	55054	ATG16 autophagy related 16-like 1 (S. cerevisiae)	ATG16L1	90	2353	FBJ murine osteosarcoma viral oncogene homolog	FOS	170	8504	peroxisomal biogenesis factor 3	PEX3
11	89849	ATG16 autophagy related 16-like 2 (S. cerevisiae)	ATG16L2	91	2308	forkhead box O1	FOXO1	171	5289	phosphoinositide-3-kinase, class 3	PIK3C3
12	23130	ATG2 autophagy related 2 homolog A (S. cerevisiae)	ATG2A	92	2309	forkhead box O3	FOXO3	172	30849	phosphoinositide-3-kinase, regulatory subunit 4	PIK3R4
13	55102	ATG2 autophagy related 2 homolog B (S. cerevisiae)	ATG2B	93	2548	glucosidase, alpha; acid	GAA	173	65018	PTEN induced putative kinase 1	PINK1
14	64422	ATG3 autophagy related 3 homolog (S. cerevisiae)	ATG3	94	11337	GABA(A) receptor-associated protein	GABARAP	174	23645	protein phosphatase 1, regulatory (inhibitor) subunit 15A	PPP1R15A
15	115201	ATG4 autophagy related 4 homolog A (S. cerevisiae)	ATG4A	95	23710	GABA(A) receptor-associated protein like 1	GABARAPL1	175	5564	protein kinase, AMP-activated, beta 1 non-catalytic subunit	PRKAB1
16	23192	ATG4 autophagy related 4 homolog B (S. cerevisiae)	ATG4B	96	11345	GABA(A) receptor-associated protein-like 2	GABARAPL2	176	5573	protein kinase, cAMP-dependent, regulatory, type I, alpha (tissue specific extinguisher 1)	PRKAR1A
17	84938	ATG4 autophagy related 4 homolog C (S. cerevisiae)	ATG4C	97	2597	glyceraldehyde-3-phosphate dehydrogenase	GAPDH	177	5580	protein kinase C, delta	PRKCD
18	84971	ATG4 autophagy related 4 homolog D (S. cerevisiae)	ATG4D	98	2773	guanine nucleotide binding protein (G protein), alpha inhibiting activity polypeptide 3	GNAI3	178	5588	protein kinase C, theta	PRKCQ
19	9474	ATG5 autophagy related 5 homolog (S. cerevisiae)	ATG5	99	10399	guanine nucleotide binding protein (G protein), beta polypeptide 2-like 1	GNB2L1	179	5728	phosphatase and tensin homolog	PTEN
20	10533	ATG7 autophagy related 7 homolog (S. cerevisiae)	ATG7	100	57120	golgi-associated PDZ and coiled-coil motif containing	GOPC	180	5753	PTK6 protein tyrosine kinase 6	PTK6
21	79065	ATG9 autophagy related 9 homolog A (S. cerevisiae)	ATG9A	101	2894	glutamate receptor, ionotropic, delta 1	GRID1	181	8766	RAB11A, member RAS oncogene family	RAB11A
22	285973	ATG9 autophagy related 9 homolog B (S. cerevisiae)	ATG9B	102	2895	glutamate receptor, ionotropic, delta 2	GRID2	182	5861	RAB1A, member RAS oncogene family	RAB1A
23	471	5-aminoimidazole-4-carboxamide ribonucleotide formyltransferase/IMP cyclohydrolase	ATIC	103	3065	histone deacetylase 1	HDAC1	183	53917	RAB24, member RAS oncogene family	RAB24
24	573	BCL2-associated athanogene	BAG1	104	10013	histone deacetylase 6	HDAC6	184	83452	RAB33B, member RAS oncogene family	RAB33B
25	9531	BCL2-associated athanogene 3	BAG3	105	9146	hepatocyte growth factor-regulated tyrosine kinase substrate	HGS	185	5868	RAB5A, member RAS oncogene family	RAB5A

26	578	BCL2-antagonist/killer 1	BAK1	106	3091	hypoxia inducible factor 1, alpha subunit (basic helix-loop-helix transcription factor)	HIF1A	186	7879	RAB7A, member RAS oncogene family	RAB7A
27	581	BCL2-associated X protein	BAX	107	3326	heat shock protein 90kDa alpha (cytosolic), class B member 1	HSP90AB1	187	5879	ras-related C3 botulinum toxin substrate 1 (rho family, small GTP binding protein Rac1)	RAC1
28	596	B-cell CLL/lymphoma 2	BCL2	108	3309	heat shock 70kDa protein 5 (glucose-regulated protein, 78kDa)	HSPA5	188	5894	v-raf-1 murine leukemia viral oncogene homolog 1	RAF1
29	598	BCL2-like 1	BCL2L1	109	3312	heat shock 70kDa protein 8	HSPA8	189	5925	retinoblastoma 1	RB1
30	8678	beclin 1, autophagy related	BECN1	110	26353	heat shock 22kDa protein 8	HSPB8	190	9821	RB1-inducible coiled-coil 1	RB1CC1
31	637	BH3 interacting domain death agonist	BID	111	3458	interferon, gamma	IFNG	191	5970	v-rel reticuloendotheliosis viral oncogene homolog A (avian)	RELA
32	332	baculoviral IAP repeat-containing 5	BIRC5	112	3551	inhibitor of kappa light polypeptide gene enhancer in B-cells, kinase beta	IKBKB	192	10287	regulator of G-protein signaling 19	RGS19
33	57448	baculoviral IAP repeat-containing 6	BIRC6	113	9641	inhibitor of kappa light polypeptide gene enhancer in B-cells, kinase epsilon	IKBKE	193	6009	Ras homolog enriched in brain	RHEB
34	662	BCL2/adenovirus E1B 19kDa interacting protein 1	BNIP1	114	11009	interleukin 24	IL24	194	6198	ribosomal protein S6 kinase, 70kDa, polypeptide 1	RPS6KB1
35	664	BCL2/adenovirus E1B 19kDa interacting protein 3	BNIP3	115	345611	immunity-related GTPase family, M	IRGM	195	57521	regulatory associated protein of MTOR, complex 1	RPTOR
36	665	BCL2/adenovirus E1B 19kDa interacting protein 3-like	BNIP3L	116	3675	integrin, alpha 3 (antigen CD49C, alpha 3 subunit of VLA-3 receptor)	ITGA3	196	56681	SAR1 homolog A (S. cerevisiae)	SAR1A
37	60673	chromosome 12 open reading frame 44	C12orf44	117	3655	integrin, alpha 6	ITGA6	197	5265	serpin peptidase inhibitor, clade A (alpha-1 antiproteinase, antitrypsin), member 1	SERPINA1
38	23591	chromosome 17 open reading frame 88	C17orf88	118	3688	integrin, beta 1 (fibronectin receptor, beta polypeptide, antigen CD29 includes MDF2, MSK12)	ITGB1	198	83667	sestrin 2	SESN2
39	10241	calcium binding and coiled-coil domain 2	CALCOCO2	119	3691	integrin, beta 4	ITGB4	199	51100	SH3-domain GRB2-like endophilin B1	SH3GLB1
40	10645	calcium/calmodulin-dependent protein kinase kinase 2, beta	CAMKK2	120	3708	inositol 1,4,5-triphosphate receptor, type 1	ITPR1	200	23411	sirtuin (silent mating type information regulation 2 homolog) 1 (S. cerevisiae)	SIRT1
41	821	calnexin	CANX	121	2548	glucosidase, alpha; acid	GAA	201	22933	sirtuin (silent mating type information regulation 2 homolog) 2 (S. cerevisiae)	SIRT2
42	823	calpain 1, (mu/I) large subunit	CAPN1	122	11337	GABA(A) receptor-associated protein	GABARAP	202	8877	sphingosine kinase 1	SPHK1
43	11132	calpain 10	CAPN10	123	23710	GABA(A) receptor-associated protein like 1	GABARAPL1	203	83985	spinster homolog 1 (Drosophila)	SPNS1
44	824	calpain 2, (m/II) large subunit	CAPN2	124	11345	GABA(A) receptor-associated protein-like 2	GABARAPL2	204	8878	sequestosome 1	SQSTM1
45	826	calpain, small subunit 1	CAPNS1	125	2597	glyceraldehyde-3-phosphate dehydrogenase	GAPDH	205	6767	suppression of tumorigenicity 13 (colon carcinoma) (Hsp70 interacting protein)	ST13
46	834	caspase 1, apoptosis-related cysteine peptidase (interleukin 1, beta, convertase)	CASP1	126	2773	guanine nucleotide binding protein (G protein), alpha inhibiting activity polypeptide 3	GNAI3	206	6794	serine/threonine kinase 11	STK11
47	836	caspase 3, apoptosis-related cysteine peptidase	CASP3	127	10399	guanine nucleotide binding protein (G protein), beta polypeptide 2-like 1	GNB2L1	207	29110	TANK-binding kinase 1	TBK1
48	837	caspase 4, apoptosis-related cysteine peptidase	CASP4	128	57120	golgi-associated PDZ and coiled-coil motif containing	GOPC	208	10548	transmembrane 9 superfamily member 1	TM9SF1
49	841	caspase 8, apoptosis-related cysteine peptidase	CASP8	129	2894	glutamate receptor, ionotropic, delta 1	GRID1	209	81671	transmembrane protein 49	TMEM49
50	6347	chemokine (C-C motif) ligand 2	CCL2	130	2895	glutamate receptor, ionotropic, delta 2	GRID2	210	157753	transmembrane protein 74	TMEM74
51	729230	chemokine (C-C motif) receptor 2	CCR2	131	9711	KIAA0226	KIAA0226	211	8743	tumor necrosis factor (ligand) superfamily, member 10	TNFSF10
52	4179	CD46 molecule, complement regulatory protein	CD46	132	9776	KIAA0652	KIAA0652	212	7157	tumor protein p53	TP53

53	1026	cyclin-dependent kinase inhibitor 1A (p21, Cip1)	CDKN1A	133	22863	KIAA0831	KIAA0831	213	58476	tumor protein p53 inducible nuclear protein 2	TP53INP2
54	1027	cyclin-dependent kinase inhibitor 1B (p27, Kip1)	CDKN1B	134	3799	kinesin family member 5B	KIF5B	214	8626	tumor protein p63	TP63
55	1029	cyclin-dependent kinase inhibitor 2A (melanoma, p16, inhibits CDK4)	CDKN2A	135	54800	kelch-like 24 (Drosophila)	KLHL24	215	7161	tumor protein p73	TP73
56	8837	CASP8 and FADD-like apoptosis regulator	CFLAR	136	3916	lysosomal-associated membrane protein 1	LAMP1	216	7248	tuberous sclerosis 1	TSC1
57	25978	chromatin modifying protein 2B	CHMP2B	137	3920	lysosomal-associated membrane protein 2	LAMP2	217	7249	tuberous sclerosis 2	TSC2
58	128866	chromatin modifying protein 4B	CHMP4B	138	84557	microtubule-associated protein 1 light chain 3 alpha	MAP1LC3A	218	286319	tumor suppressor candidate 1	TUSC1
59	1201	ceroid-lipofuscinosis, neuronal 3	CLN3	139	81631	microtubule-associated protein 1 light chain 3 beta	MAP1LC3B	219	8408	unc-51-like kinase 1 (C. elegans)	ULK1
60	1508	cathepsin B	CTSB	140	440738	microtubule-associated protein 1 light chain 3 gamma	MAP1LC3C	220	9706	unc-51-like kinase 2 (C. elegans)	ULK2
61	1509	cathepsin D	CTSD	141	5609	mitogen-activated protein kinase kinase 7	MAP2K7	221	25989	unc-51-like kinase 3 (C. elegans)	ULK3
62	1514	cathepsin L1	CTSL1	142	5594	mitogen-activated protein kinase 1	MAPK1	222	9100	ubiquitin specific peptidase 10	USP10
63	6376	chemokine (C-X3-C motif) ligand 1	CX3CL1	143	5595	mitogen-activated protein kinase 3	MAPK3	223	7405	UV radiation resistance associated gene	UVRAG
64	7852	chemokine (C-X-C motif) receptor 4	CXCR4	144	5599	mitogen-activated protein kinase 8	MAPK8	224	9341	vesicle-associated membrane protein 3 (cellubrevin)	VAMP3
65	1612	death-associated protein kinase 1	DAPK1	145	9479	mitogen-activated protein kinase 8 interacting protein 1	MAPK8IP1	225	6845	vesicle-associated membrane protein 7	VAMP7
66	23604	death-associated protein kinase 2	DAPK2	146	5601	mitogen-activated protein kinase 9	MAPK9	226	7422	vascular endothelial growth factor A	VEGFA
67	1649	DNA-damage-inducible transcript 3	DDIT3	147	51360	membrane-bound transcription factor peptidase, site 2	MBTPS2	227	23001	WD repeat and FYVE domain containing 3	WDFY3
68	9077	DIRAS family, GTP-binding RAS-like 3	DIRAS3	148	64223	MTOR associated protein, LST8 homolog (S. cerevisiae)	MLST8	228	11152	WD repeat domain 45	WDR45
69	10395	deleted in liver cancer 1	DLC1	149	64419	myotubularin related protein 14	MTMR14	229	56270	WDR45-like	WDR45L
70	3337	DnaJ (Hsp40) homolog, subfamily B, member 1	DNAJB1	150	2475	mechanistic target of rapamycin (serine/threonine kinase)	MTOR	230	55062	WD repeat domain, phosphoinositide interacting 1	WIPI1
71	4189	DnaJ (Hsp40) homolog, subfamily B, member 9	DNAJB9	151	4609	v-myc myelocytomatosis viral oncogene homolog (avian)	MYC	231	26100	WD repeat domain, phosphoinositide interacting 2	WIPI2
72	55332	DNA-damage regulated autophagy modulator 1	DRAM1	152	92345	nuclear assembly factor 1 homolog (S. cerevisiae)	NAF1	232	53349	zinc finger, FYVE domain containing 1	ZFYVE1
73	9695	ER degradation enhancer, mannosidase alpha-like 1	EDEM1	153	10135	nicotinamide phosphoribosyltransferase	NAMPT				
74	1938	eukaryotic translation elongation factor 2	EEF2	154	4077	neighbor of BRCA1 gene 1	NBR1				
75	29904	eukaryotic elongation factor-2 kinase	EEF2K	155	10787	NCK-associated protein 1	NCKAP1				
76	1956	epidermal growth factor receptor (erythroblastic leukemia viral (v-erb-b) oncogene homolog, avian)	EGFR	156	4780	nuclear factor (erythroid-derived 2)-like 2	NFE2L2				
77	5610	eukaryotic translation initiation factor 2-alpha kinase 2	EIF2AK2	157	4790	nuclear factor of kappa light polypeptide gene enhancer in B-cells 1	NFKB1				
78	9451	eukaryotic translation initiation factor 2-alpha kinase 3	EIF2AK3	158	159296	NK2 transcription factor related, locus 3 (Drosophila)	NKX2-3				
79	1965	eukaryotic translation initiation factor 2, subunit 1 alpha, 35kDa	EIF2S1	159	58484	NLR family, CARD domain containing 4	NLRC4				
80	1978	eukaryotic translation initiation factor 4E binding protein 1	EIF4EBP1	160	4864	Niemann-Pick disease, type C1	NPC1				
